# Direct Ink Writing Technology (3D Printing) of Graphene-Based Ceramic Nanocomposites: A Review

**DOI:** 10.3390/nano10071300

**Published:** 2020-07-02

**Authors:** Nestor Washington Solís Pinargote, Anton Smirnov, Nikita Peretyagin, Anton Seleznev, Pavel Peretyagin

**Affiliations:** Spark Plasma Sintering Research Laboratory, Moscow State University of Technology “STANKIN”, Vadkovsky per. 1, Moscow 127055, Russia; n.peretyagin@stankin.ru (N.P.); a.seleznev@stankin.ru (A.S.); p.peretyagin@stankin.ru (P.P.)

**Keywords:** additive manufacturing, graphene oxide, graphene-based paste, direct ink writing, ceramic nanocomposites

## Abstract

In the present work, the state of the art of the most common additive manufacturing (AM) technologies used for the manufacturing of complex shape structures of graphene-based ceramic nanocomposites, ceramic and graphene-based parts is explained. A brief overview of the AM processes for ceramic, which are grouped by the type of feedstock used in each technology, is presented. The main technical factors that affect the quality of the final product were reviewed. The AM processes used for 3D printing of graphene-based materials are described in more detail; moreover, some studies in a wide range of applications related to these AM techniques are cited. Furthermore, different feedstock formulations and their corresponding rheological behavior were explained. Additionally, the most important works about the fabrication of composites using graphene-based ceramic pastes by Direct Ink Writing (DIW) are disclosed in detail and illustrated with representative examples. Various examples of the most relevant approaches for the manufacturing of graphene-based ceramic nanocomposites by DIW are provided.

## 1. Introduction

A ceramic is a nonmetallic, inorganic solid [1], which has exceptional and diverse physical and chemical properties that characterize it as a multipurpose material. Typical properties that can be found in ceramics materials are ultra-high-temperature ability, excellent wear resistance, great hardness and mechanical strength, high melting points, good thermal stability, and chemical inertness, low density, and low electrical and thermal conductivity. Thanks to these properties, ceramics are used in multifunctional applications such as biomedical engineering, electronics, aerospace, chemical industry, and machinery [2]. Note that the advantage of ceramics over other materials is the ability to obtain predetermined characteristics by changing the raw materials composition and the production technology [3,4,5,6,7,8]. Commonly, raw materials are composed by mixtures of ceramic powders with or without binders and additives, and these mixtures are used to form green bodies with desired simple shape by different forming methods as dry pressing, slip casting, injection molding, gel casting, tape casting, extrusion and others [9,10]. After forming, the green parts are very soft; therefore, it is necessary to apply heat upon them to get a dense product by sintering. Sintering can be defined as a thermal process at higher temperatures with or without pressure for compacting and forming a solid structure via mass transport events that often occur on the diffusional processes [10]. Although traditional methods of ceramic forming are well-studied and widespread, they have several drawbacks such as high cost, long processing times and the impossibility of producing pieces with interconnected holes or with highly complex shapes. In addition, for obtaining a sintered ceramic part with high surface quality and accuracy, mechanical post-processing work is necessary. This post-process work is expensive and time-consuming due to the natural high hardness and brittleness of ceramics materials [2,11].

Over the past 30 years, new technologies for processing materials called additive manufacturing (AM) have been developed rapidly and they are being introduced more and more every day in a wide range of fields thanks to their ability to produce, in a very fast and cheap way, complex 3d parts by adding material instead of cutting it away. AM, also known as three-dimensional (3D) printing technologies [12], can be explained as a technique of blending materials by either fusion, binding, or solidifying materials such as liquid resin and different powders materials. These technologies build a part in a precisely adding material layer-by-layer fashion using 3D computer-aided design (CAD) modeling [13], and their advantages include design freedom, low-quantity economy, material efficiency, reduced assembly and predictable production.

AM involves a group of advanced manufacturing technologies that allow the flexible production of precise structures with highly complex shapes, which are complicated to manufacture using conventional methods like machining or casting [14].

In 2015, the International Organization for Standardization Technical Committee (ISO/TC 261) on AM together with the American Society for Testing Materials (ASTM) Committee F42 released a new International Standard ISO/ASTM 52900:2015 in which the terms used in AM are established and defined [15]. In this standard, the AM technologies have been classified into groups taking into consideration the feedstock type, the deposition technique, and the fusing or solidification way of material. Table 1 categorizes the most popular AM technologies in the industry today into the following groups: vat photopolymerization, material jetting, binder jetting, powder bed fusion, direct energy deposition, sheet lamination, and material extrusion.

The main differences between each of the categories mentioned above can be summarized as follows [16]:

- In vat photopolymerization the 3D object is created layer by layer thanks to the curing of a liquid photopolymer resin under the influence of an ultraviolet (UV) light. The liquid photopolymer is held in a vat with a built support submerged near the surface of the resin. Then the UV light is directed to the resin surface following a determined path thus allowing a selective local polymerization of the liquid photopolymer. After that, the built support is re-submerged into the resin and the process is repeated until the 3D object is fully obtained;
- The material jetting principle is used to create a solid 3D object layer by layer from droplets, which are mainly composed of liquid photopolymer resin, that are selectively sprayed by an inkjet-style printhead and immediately cured thanks to the expose of a UV light. Commonly, technologies that work under this principle are compared to the two-dimensional (2D) inkjet printing, which deposits only a single layer of ink droplets;
- In the technologies that operate under the principles of binder jetting a liquid binder, which is selectively deposited by drops onto a powder-based material using an inkjet-style print head, is utilized in order to produce a solid 3D object layer by layer. During the process, alternate layers of powder material and binding material are depositing as follow: powder particles are spread over a built support using a roller while the print head deposits the liquid binder, which acts as a glue between powder particles and layers, on top of the powder bed; after that, the built support is lowered by the model’s layer thickness and then the process is repeated until the 3D object is formed;
- The powder bed fusion category utilizes an energy source that allows the local sintering or melting between the particles of a powder material for the forming of a solid 3D object layer by layer. The energy sources can be lasers or electron beams depending on the using material powder. The electron beam is necessary for metals, while lasers are required for polymers. The forming part process is very similar to binder jetting: powder particles are spread over a built support using a roller while the energy source fuses the first layer; after that, the built support is lowered by the model’s layer thickness and a new layer of powder is spread across the previous layer repeating the process until the 3D object is formed;
- In direct energy deposition the 3D object is created layer by layer thanks to the directly melting of build-material and deposing them on the workpiece using a focused thermal energy source such as laser, electron beam or plasma arc. This principle can be applied for a wide kind of materials such as polymers, ceramics, and metal framework composites; however, it is predominantly used for wire and powder metals, which explains why this technology is often called metal deposition. Direct energy deposition utilizes a nozzle mounted on a multi-axis arm can move freely in any direction of the x, y and z-axes that deposits melted material onto the predetermined workpiece surface, where it is automatically solidified;
- In sheet lamination a 3D part is created by bonding together, layer-by-layer, thin sheets of material (usually supplied via a system of feed rollers), which is then cutting into a final 3D object. In the process, the sheet material is positioned on the cutting bed and then it is bonded over the previous layer using any suitable sticky method; after that, the required shape is cut by laser or knife and the process is repeated until the 3D object is formed. Laminated object manufacturing (LOM) and ultrasonic consolidation (UC) are both examples of sheet lamination techniques;
- Material extrusion is a category of AM, in which the 3D object is formed by a layer by layer selective deposition of the extruded build-material through a nozzle in a continuous stream. In material extrusion, the layers are built when the nozzle deposits a viscoelastic material where it is required. The following layers are added on top of previous layers and bonded upon deposition as the material shows viscoelastic behavior. In the last past years, this technology became popular in the world for its use in 3D printers. Direct Ink Writing (DIW) and Fused deposition modeling (FDM) are the two common technologies that operate under the principles of material extrusion. However, in the last past years, a new AM technique named Pyro-EHD Tethered Electrospinning (TPES) that is based on electrohydrodynamic processes and can be related to material extrusion category became more and more popular [17,18,19].

The introduction of AM into the ceramic forming process proposes a powerful way of producing complex 3D parts. However, despite the wide variety of AM technologies, only a few of them can be implemented for printing ceramic parts. Among such technologies, the so-called Direct Ink Writing (DIW) offer greater versatility and particular suitability for the fabrication of ceramic parts [20]. DIW, also referred to as Robocasting [21], is an extrusion-based technique used in 3D printing in which new materials can be implemented most economically and flexibly [22,23]. The main requirement of this technology is the use of pastes with controlled rheological behavior that allows them to be able to be extruded into filaments capable of maintaining their shape and not collapsing during the 3D object forming process [24]. The required rheological characteristics can be achieved through the correct selection of the number of components, solid-phase parameters and the additives used [25]. 

In the industrial manufacture of ceramic parts, it is very common the use of slurry that contains various additives, such as plasticizers, dispersants, surfactants, binders, defoamers, lubricants, etc., which in many occasions produce the formation of defects during sintering [26]. These defects can be related to the evaporation of the aforementioned additives that leads to volumetric shrinkage and crack formation, which considerably reduces the mechanical properties of the part [27].

Some years ago, the use of chemically modified graphene (in other words, graphene oxide (GO)) has been proposed with aim of prepare an aqueous paste without any additive for the 3D printing of graphene-based heaters [28]. Later, García-Tunñoón et al. formulated free additives pastes of diverse materials, based only on the use of GO as a dispersant, rheological modifier, and binder [29]. It was possible, because GO has a great similarity to clay, including its viscoelastic behavior. Clay has exceptional chemistry and structure that permit the design of water-based suspensions for shaping with excellent viscoelastic behavior in a procedure that cannot be done with any other natural material [30]. The special combination of surface chemistry and the structure of GO sheets in contact with water under special conditions allow the preparation of a very stable GO suspension with viscoelastic behavior comparable to clay [29,31]. In addition, GO as an oxide can be homogeneously dispersed in water, and, consequently, mixtures of graphene oxide with any ceramic oxide can be processed following conventional ceramic processing routes [32,33,34,35]. Therefore, the implementation of GO to obtain ceramic pastes without additives for their use in AM opens up new possibilities for obtaining complex parts with the help of robocasting technology.

The aim of the present work is to explain the state of the art of the most common AM technologies used for the manufacturing of complex shape structures of both ceramic and graphene-based parts; and, disclose the most important works about the fabrication of composites using graphene-based ceramic pastes by DIW. Where it was necessary, an explanation of important aspects of AM techniques for ceramic or graphene-based materials will be presented. Moreover, overviews of diverse examples of graphene-based pastes for DIW are given. In where it was possible, historical facts of diverse AM techniques were explained. 

Our article is structured as follows: Section 2 explains a short review of the AM processes for ceramic, which are grouped by the type of feedstock used in each technology; besides, we tried to summarize their principles and applications, and to provide their most important historical facts. Section 3 describes in more detail the AM processes used for 3D printing of graphene-based materials and cites some studies in a wide range of applications related to these AM techniques. Different feedstock formulations and their corresponding rheological behavior were explained. Section 4 is focused on the more actual developments on direct ink writing by the use of graphene-based ceramic pastes. We provide some examples of the most relevant approaches for the manufacturing of graphene-based ceramic composites by DIW. Finally, in Section 5, a summary of this work is described.

## 2. Additive Manufacturing Processes for Ceramic and Their Principles

The beginning of AM technologies dates back to July 16, 1984, when André J.C., Le Mehauté A. and De Witte O. filed a patent at Cilas Alcatel [36], in which the stereolithography process was proposed. Three weeks later, on August 8, 1984, Charles W. Hull filed his patent at UVP, Inc. and coined the term “stereolithography” (SLA) [37]. After that, the development of AM was followed by technologies as powder bed fusion, fused deposition modelling (FDM), inkjet printing and others [38]. However, it was only in the 1990s when the first reports of 3D printing of ceramic materials appeared [39].

Today, there is a wide variety of AM technologies used for different types of materials. Table 1 lists the most popular manufacturing additive technologies in the industry, and the possible types of feedstock that can be used in each technology [17]. From this, it is easy to appreciate that not all AM technologies are suitable for the processing of ceramic materials.

**Figure 1:**
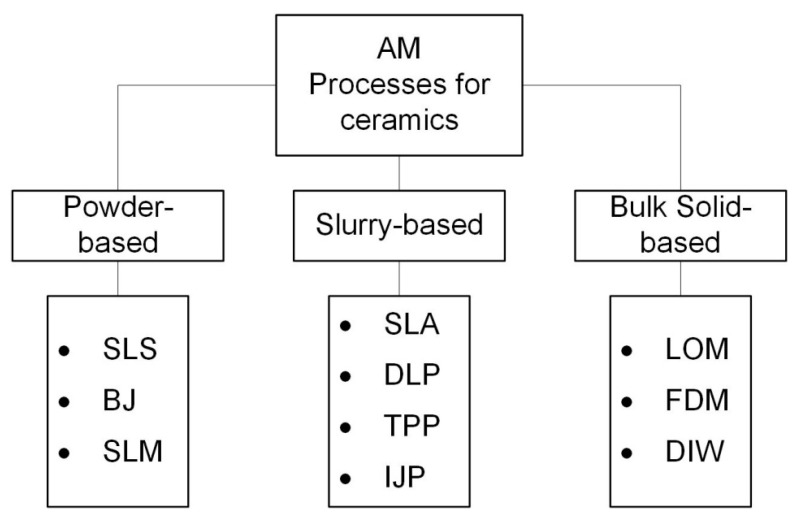
Classification of AM technologies for ceramics by the type of feedstock used: SLS–Selective Laser Sintering; BJ–Binder Jetting; SLM–Selective Laser Melting; SLA–Stereolithography; DLP–Digital Light Processing; TPP–Two-Photon Polymerization; IJP–Ink Jet printing; LOM–Laminated Object Manufacturing; FDM–Fused Deposition Modeling and DIW–Direct Ink Writing.

In 1991, Professor J.P. Kruth first organized the AM processes according to the form of the used material before printing [40], and his classifications were: powder, solid and liquid-based techniques. Using this principle, it is possible to group the AM technologies for ceramic materials into powder-based, bulk solid-based, and slurry-based, as shown in Figure 1.

In addition, it should be noted that the traditional ceramic forming technology such as dry and isostatic pressing, sliding and tape casting, etc. have some limitations. They cannot be used for parts with complex shapes (with inner holes, sharp corners, etc.), and which require high precision. Moreover, these forming methods need the manufacture of molds and post-processing, which is time-consuming and costly [41,42]. Meanwhile, AM technologies such as powder-, slurry- and bulk solid-based techniques are promising methods for producing near net complex shape parts and consequently allows for saving time and reducing production costs of required products in comparison with traditional forming methods.

### 2.1. Powder-Based Technologies

In this group of AM technologies for ceramic materials, powder beds are used. During the additive process, the ceramic powder can be bonded by melting, sintering, or a binder agent, depending on the type of technology used. Among the AM technologies that utilize powder beds, for ceramic materials three of them are the most important in the industry: selective laser sintering (SLS), selective laser melting (SLM) and binder jetting (BJ).

#### 2.1.1. Selective Laser Sintering (SLS)

**Figure 2:**
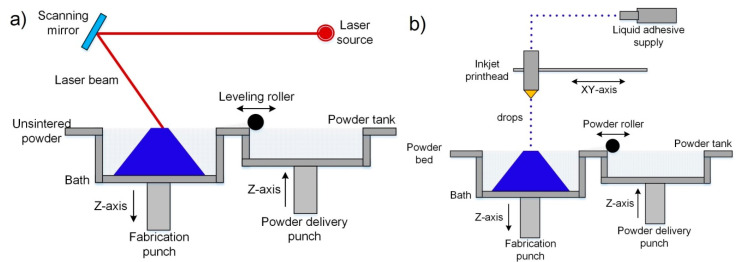
Schematic diagrams of powder-based additive manufacturing (AM) technologies main methods: (**a**) SLS and SLM; (**b**) BJ. Adapted from [44], with permission from Wiley-VCH Verlag GmbH and Co. KGaA, Weinheim, 2018.

This method was first reported by Deckard when he filed a patent in 1986 at the University of Texas, and the main goal of this technology was to fabricate wax objects for their use in investment casting in order to obtain metallic prototypes [43]. The SLS process belongs to the category “powder bed fusion”, and utilizes a high power laser beam as a thermal source for the local sintering of a thin layer in the powder material surface. When the powder is heated enough by a laser beam a diffusion process takes place between particles in the powder, which leads to the densification of the material. The part forming is a layer by layer process. In this way, when a layer is sintered, the powder bed moves down by an elevator system, a new powder layer is distributed onto the previous surface using a spreading system, and then a new cycle of sintering, descent, and spreading is repeated until the final part is formed. Figure 2a, [44], shows a schematic diagram of the SLS process.

The powder bed can be a single material with a low melting point, or a mixture of a high melting point material together with inorganic or organic binders which may need debinding by high-temperature heat treatment to get the final part [45,46,47]. The SLS process must be carried out in vacuum or inert atmospheres such as argon and nitrogen to avoid the oxidation of the binder during sintering. An advantage of this method is that it does not need the creation of additional supports, since the low sintering temperatures do not produce internal stresses that can deform the fabricated part. 

Although the laser beam can generate a high local temperature, it is not enough for the sintering of the ceramic materials. Thus, it also requires a long dwell time of the laser beam exposition for ceramics. A solution can be to use a binder material with a lower melting point to coat or mix the ceramics particles with it. This approach was used by Lakshminarayan et al. in 1990, when they reported the first complex shape 3D part obtained by SLS [48].

Moreover, the high porosity remaining in the final parts and the high shrinkage are two major problems related to SLS of ceramics [49]. On the other hand, the benefit of this method is that no support structures have to be deliberately fabricated for overhanging regions because the parts are among the loose powder in the bed at all times. Thus, the properties of the parts produced by SLS are affected by several factors involved primarily with the feedstock materials and laser–material interactions should be taken into account during the fabrication process.

#### 2.1.2. Binder Jetting (BJ)

Binder jetting is a process invented 1989 by Sachs et al. when they filed a patent for it at Massachusetts Institute of Technology [50], and thanks to its main characteristics, it belongs to the category “Binder jetting”. The principal objective of this technology was to rapidly-produce of parts from a larger variety of materials as plastics, metals and ceramics [14]. The BJ process utilizes an organic binder solution that is dropped into a powder bed for the gluing of particles in the surface by a printhead in determined paths. The scheme of the BJ process is shown in Figure 2b [44].

The first report about the application of the BJ process on ceramic materials was made by Sachs et al., in 1992, when they used a matrix of alumina and silicon carbide with colloidal silica as a binder [51]. Any ceramic powders, can be used to prepare ceramic preforms, and color printing all of these provide the benefit of BJ with regards to other fabrication methods. However, it must be mentioned that the binder agent, powder material, equipment, printing parameters, and post-treatment influence on the strength of ceramic parts and their accuracy. In BJ, like SLS, the part forming is one layer at a time. When a layer is consolidated, a new powder layer is distributed onto the previous surface using a spreading system, and then a new cycle of gluing and spreading is repeated until the final 3D part is formed. After the part was fabricated, the organic binder must be removed by sintering to obtain the desired mechanical properties. The debinding generally produces shrinkage and porous in the part that depends on the binder percentage in it. The mechanical properties are negatively affected by the amount of porosity in the model. Despite this, BJ is a good method used in biomedical fields for the ceramic scaffold production, thanks to porosities that facilitates the cell cultivation purposes.

In the coming years, BJ must become a widespread technique for making 3D ceramic printed parts if the difficulties with their strength and accuracy can be solved producing a preform of any complex form that does not shrink after post-treatment.

#### 2.1.3. Selective Laser Melting (SLM) 

The SLM method was first reported in Germany by Wilhelm Meiners et al. when they filed the patent DE 19649865 in 1996 [52]. This process belongs to the category “powder bed fusion” because it is considered a variant of SLS that uses a laser beam with much higher energy densities for the local sintering of material [16]. The presence of a more power thermal source allows the sintering process to be carried out in a single step (without any postprocessing) and not using second phases with low melting temperatures as binders. Figure 2a [44], shows the schematic of the SLM conceptual principle, which is the same as SLS.

Throughout time, this technique remains very popular among AM technologies for metals thanks to the ability to manufacture parts quickly and efficiently [53]. Moreover, SLM allows the formation of dense parts since the powder particle is completely melted into the liquid phase ensuring rapid densification without the need for a debinding process.

This technique can be included in the manufacture of ceramic parts, but it implies the need to achieve the complete melt of ceramic powder [54]. Since ceramic materials have very high melting points their complete melt in SLM represents a serious technological problem that limits the application of this technology in ceramic production [55]. Despite this, when the laser sweeps across the powder bed, different areas of the parts will experience high-temperature fluctuation, creating high thermal stresses. Combined with the low ductility of ceramic materials, cracks can form [56]. The correct control and management of the energy applied to the powder are of great importance since the application of low energy levels leads to the incomplete melting of the material, and on the other hand, under the action of a high energy level, the powder material is spattered around it [57].

However, ceramic parts with a 100% density were produced by SLM with complete melting of the material and without any post-processing [58]. In this work, the authors utilized a eutectic mixture of Al_2_O_3_ ± ZrO_2_ to lower the melting point of the material, which was ultrahigh preheated above 1600 °C in order to prevent the possible crack formation during the build-up process. This approach made the process very complicated, but it demonstrates that SLM is the only perspective method that promises to get ceramic parts with full density directly after its use.

As in other methods, the SLM-produced ceramic part quality is affected by several factors, like fabrication parameters, raw material properties, post-processing and the interaction of the energy source and the materials.

### 2.2. Slurry-Based Technologies

This group of AM technologies for ceramic materials involves the methods that use feedstock, in the form of inks. These inks are liquid systems dispersed with fine ceramic particles. During the additive process, the ceramic parts can be printed by photopolymerization or material jetting, depending on the type of technology used. Among the AM technologies that utilize inks, for ceramic materials four of them are the most important in the industry [13]: stereolithography (SLA), digital light processing (DLP), two-photon polymerization (TPP) and inkjet printing (IJP). Note that slurry-based technologies utilize inks, which are mixtures of polymer with ceramics particles, that have low-viscosities in the range of mPa·s and with a low ceramic loading (up to 30 vol%).

#### 2.2.1. Stereolithography (SLA)

**Figure 3:**
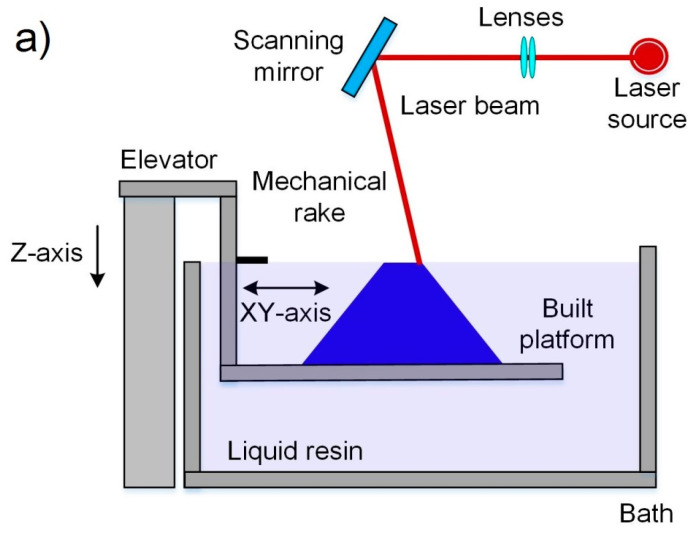

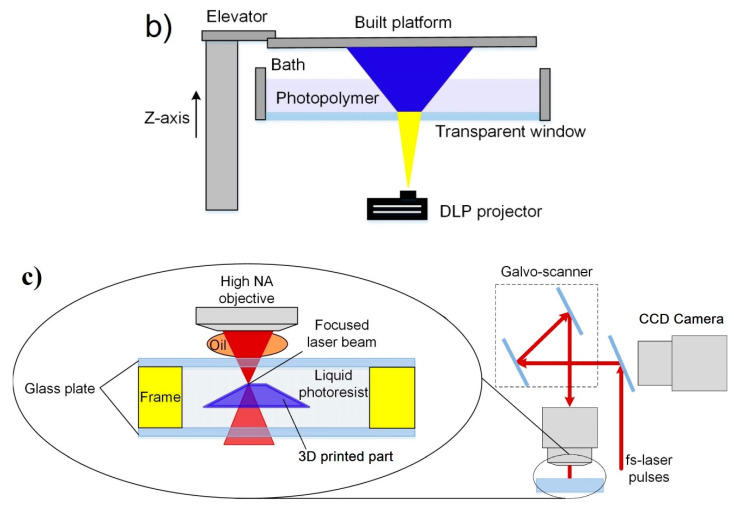
Schematic diagrams of slurry-based AM technologies main methods: (**a**) SLA; (**b**) DLP; (**c**) two-photon polymerization (TPP). Figure 3**a**,**b** adapted from [44], with permission from Wiley-VCH Verlag GmbH & Co. KGaA, Weinheim, 2018.

Stereolithography imposed the start of the additive manufacturing era in the mid-1980s when a patent about this method was filed [37]. The SLA process utilizes a light source for the curing of a liquid photopolymer tiny layer in a vat consisting mainly of photopolymerizable monomer with other additives in very small amounts [59]. For this reason, this process is included in the category “Vat photopolymerization”. Commonly, in this method, an ultraviolet (UV) light source is used. This UV light scans the surface of the photopolymer, following a controlled path, thanks to the help of motorized mirrors. A schematic diagram of the SLA is shown in Figure 3a [44]. When a photopolymer interacts with the UV beam, the light activates a series of reactions that are known as polymerization. Polymerization is a process in which monomers crosslink to form polymers, that lead to a phase change from liquid to solid resin [60]. In SLA the part forming is a layer by layer process. In this way, when a part layer is polymerized its support is lowered a distance equal to the thickness of the new layer by an elevator system. After that, a new cycle of polymerization and lowering is repeated until the final part is formed. Figure 3a, [44], shows a scheme of the SLA process.

The first study of SLA with ceramic particles was reported in 1994 [61]. In that work, the authors used slurry with a very high concentration of particles, about 65 vol%. Commonly, the SLA of ceramics uses fine ceramic particles that are dispersed in the liquid photopolymer [62,63]. In this case, the photopolymer is cured by light irradiation creating a solid resin with a uniform distribution of ceramic particles. After the fabrication of a 3D part, this green body must be processed at high temperatures to eliminate the organic compounds and to rich a higher density. 

As a slurry-based AM technology, the feedstock must have certain essential requirements to obtain a good result during the polymerization. First of all, there is the need for a suspension with a high and homogeneous distribution of the ceramic particles. This suspension must have a good rheological behavior that includes an optimum viscosity in each case of material and good stability of the suspension over time [16].

The advantages of this method are the ability to build intricate geometries, accommodate large build areas, smooth surface, accuracy, and high resolution [64]. However, this technique is still limited to ceramic materials, which are no able to absorb UV radiation. Indeed, the introduction of small (<1 µm) ceramic particles in a curable monomer increase a level of complexity in comparison to the classical fabrication of pure polymeric materials. The main difficulty is related to the scattering phenomena, which decrease the cure depth and then increase rise the processing time. Besides, this phenomenon decreases the dimensional resolution of the printed part.

#### 2.2.2. Digital Light Processing (DLP)

The DLP process is an improved version of SLA, in which a UV light source is not used for the solidification of the photopolymer. In this process, a white light source is used to project the cross-sections of the 3D part, one projection at a time, with the help of a digital mask [65]. The idea of this process was invented by Nakamoto and Yamaguchi in 1996 when they used a physical mask instead of a digital projector [66]. This process, as well as the SLA, also belongs to the category “vat photopolymerization”.

During the DLP process, the liquid photopolymer is exposed to the direct projection of the sectioned figure of the model for its polymerization with the help of the digital mask [67]. Then, the solid surface changes its vertical position, thanks to the help of an elevation system, changing the planar focus for the projection and formation of the new layers with the required sectioned shape of the 3D model. This process is repeated until the part is completely fabricated. Figure 3b, [44], shows a scheme of the DLP process.

DLP, as opposed to to SLA, is a much faster and cheaper process that has a very high-resolution thanks to the easy choice of the optimal size of the projected pixel [68]. These advantages make it a very attractive process for the ceramic industry [69]. The use of this method in the production of ceramic pieces has acquired a great impact, and to this day it is one of the most used techniques thanks to the possibility of obtaining pieces with high densities and hardness including a high quality of the surface [70]. The DLP technology used exclusively for ceramic materials is generally marketed under the name of lithography-based ceramic manufacturing (LCM) [71]. Generally, this method is used to fabricate heat exchangers [72], meta-material structures and cellular ceramic structures with very small feature sizes [73,74].

At the same time, the main disadvantages of this technology compared to SLA are: parts cannot be left out in the sun or they will degrade; parts overall have worse mechanical properties—they break or crack more easily and are at risk of deteriorating over time; resins are expensive, and the regular replacements of resin tanks and occasionally print platforms also adds up.

#### 2.2.3. Two-Photon Polymerization (TPP)

Two-photon polymerization (TPP) is an AM technology related to the “vat photopolymerization” group in which a multiphoton polymerization-based process is used. The principal purpose of this method is to utilize the nanoscale fabrication of parts for their application in different areas such as nanoelectronics, nanomechanics, and nanobiomedicine. The first work-related with the TPP field was carried out by Wu et al. in 1992 to get high-aspect-ratio trenches of simple shapes [75]. Later, this process was improved and applied to fabricate complex forms, as was shown in the work of Maruo et al. in 1997 [76].

The polymerization process in TPP is based on the interaction of two or more photons from a laser with a specific wavelength when it focuses on a specific point within a liquid photopolymer. A schematic diagram of the TPP is shown in Figure 3c. After polymerization of a certain volume of material, the focus point of the laser is moved for the formation of the following layers. The final resolution and the quality of the surface obtained depend directly on the precise positioning and control of the size of the focal point [77].

The advantages of TPP are mainly related to the possibility of polymerizing tiny volumes at high speed within the photopolymer which gives the possibility of manufacturing microstructures with lateral feature sizes in the order of nanometers [78,79]. The use of a technology with this type of characteristics for the ceramic part production with complex shapes opens up new and interesting possibilities for this industry.

The first work on TPP for the obtaining of ceramic parts was published by Pham et al. [80]. In that work, a SiCN woodpile nano- and micro-ceramic microstructures of submicron resolution was created.

This technology has a series of restrictions that limit its use and among these, we can define: very expensive, time-consuming, and challenging for complex structures. Furthermore, the working principle of TPP allows only the use of transparent polymers; this means that the opaque polymers that were usually used for SLA and DLP processes are not applicable for TPP. It should be noted that this process can produce only very small parts on the order of a few microns and due to the high precision of the process; it also takes a longer amount of time to completely manufacture a part.

#### 2.2.4. Inkjet Printing (IJP)

**Figure 4:**
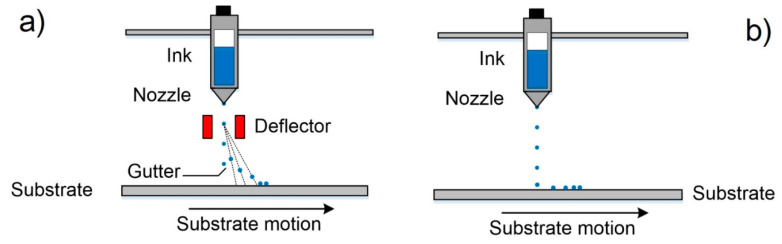
Schematic diagrams of printing methods used in Inkjet printing: (**a**) continuous inkjet (CIJ); (**b**) drop-on-demand (DOD).

Inkjet printing, sometimes known as material jetting, is a non-contact method of AM mainly created to obtain two-dimensional patterns inspired by a technology developed in the 1950s [81]. The IJP process is based on the controllable dispensing of liquid-phase materials by the use of micrometer-sized printhead nozzles [82], which is the reason why this technology is related to the material jetting category. The liquid-phase material, also known as Ink, is dispensed onto a surface by droplets in a specified pattern in which the ink drying occurs forming a thin layer of the ink residue. After that, new layers can be placed on top of each other to form a multilayer 3D object. The IJP processes can use one of the main two methods of ink dispensing: continuous inkjet (CIJ), or drop-on-demand (DOD) printing [83]. Figure 4 shows the printing methods used in IJP.

The CIJ method involves the production of a stream of drops jetting out through a controllable micronozzle [65]. Then, the formed droplets pass through an electrostatic field that influences them and deflects their trajectories to print on a substrate or allows them to follow their movement towards a collector for reuse. In this way, only small quantities of droplets are used for printing, and the largest number of drops is recycled, i.e., CIJ is a non-economical material process. The DOD method produces ink droplets when and where it is required, thus this method is more economical than CIJ. Moreover, the small size of droplets and the high positioning accuracy make it more ideal for 3D printing [84]. In DOD the droplets can be formed by the piezoelectric effect, thermal excitation or pyro-electric effect in the printing nozzle head [84,85,86]. Piezoelectric DOD utilizes a piezoelectric element located in the fluid chamber near the nozzle head for the droplet formation [87]. In this case, the droplet is created and ejected by a generating pressure pulse that forces the ink to leave the nozzle head [84]. The pressure pulse is formed thanks to the piezoelectric element deformation under the application of a voltage. When a droplet is ejected, it falls by gravity, and then it is impregnated in the subtracted thanks to the momentum obtained during its movement [14]. After that, occurs the solvent evaporation from the impregnated droplet creating a small layer made up of the ink residue. In thermal DOD process, sometimes named as bubble jet printing [14], a thermal excitation for the formation and ejecting of the droplets is used [15]. The thermal excitation is generated when a current cross through a resistive element located in the fluid chamber near the nozzle head and that directly contacts with the ink. When the heater element temperature reaches the ink’s boiling point, rapid ink vaporization is occurred creating a bubble that expands in the fluid chamber, forcing a droplet out of the nozzle. The bubble formation is followed by a very fast collapse when the pulse current source is “switched off”. 

Different types of materials, such as metals or polymers for electronic patterning [88], paste for electronics soldering and cells for restoration in tissue engineering [89,90], were used in IJP. However, the IJP method is limited only to the production of miniaturized parts due to the low ink volume used in each droplet. The obtaining of a solid ceramic part after an IJP process involves the drying and sintering post-processing of the printed part. 

Thanks to the development of computational technologies, the increase in positioning precision and advances in the 3D printing field, the use of the IJP method has been achieved for ceramic materials dispersed in liquid solvents. The first report of IJP with ceramic materials dates back to 1995 when Blazdell et al. used ceramic inks (ZrO_2_ and TiO_2_) with a volumetric fraction of 5% [91]. After that, different researches groups improved the IJP and the feedstock preparation for obtaining materials with ceramic loading until 20% [92].

### 2.3. Bulk Solid-Based

This group of AM technologies for ceramic materials involves the methods that use material sheets, semi-molten or semi-liquid systems in which fine ceramic particles are well dispersed as feedstock. Note that semi-molten and semi-liquid systems, commonly of ceramic/polymer mixtures, have a high-viscosity in the range of some Pa·s like pastes that have a higher content of ceramic (up to 60 vol%). In this group, some AM technologies that belong to different categories such as Sheet lamination or Material Extrusion will be considered. Among the AM technologies that utilize solid feedstock for ceramic materials are laminated object manufacturing (LOM), Fused Deposition Modeling (FDM), and direct ink writing (DIW) that are the most important in the industry [13].

#### 2.3.1. Laminated Object Manufacturing (LOM)

**Figure 5:**
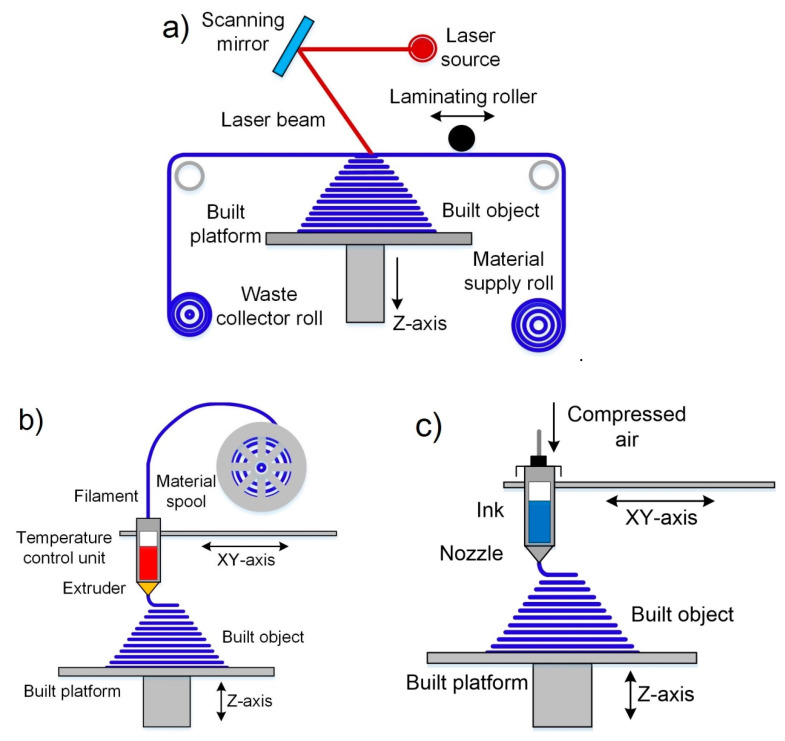
Schematic diagrams of bulk solid-based AM technology main methods: (**a**) LOM; (**b**) FDM; (**c**) direct ink writing (DIW). Figure 5**a**,**b** adapted from [44], with permission from Wiley-VCH Verlag GmbH & Co. KGaA, Weinheim, 2018.

Laminated object manufacturing is a process related to the Sheet lamination group and it was mainly developed for the fabrication of metal, paper or plastic parts. The first report of this method was in 1984 when Kunieda published his work “Manufacturing of High Cycle and High Precision Injection Molds by Diffusion Bonding of Laminated Thin Metal Sheets” in which molds for injection molding of plastics were obtained by laminating metal sheets cut by laser [93]. As said before, the concept of this method is to utilize, generally obtained by laser cutting, tiny pre-patterned material sheets that are superposed on top of each other and attached by either using a heat source or adhesives to form 3D parts [94]. The type of the used joining process is depending on the raw material. For instance, sheets of metal materials are bonding by the use of ultrasonic processes that produce local heating of the sheets creating a very strong bond [95]. For paper sheets, an adhesive material, as glue, is used. In the case of polymer materials, the use of heat and pressure is necessary [96]. Generally, this method needs a machining post-process to reach the final shape, surface quality and accuracy of the part. Figure 5a, [44], depicts the process scheme for LOM.

Laminated object manufacturing exhibit several advantages such as low process and machine cost, high volumetric build rate, low material requirement, high surface finish and the ability to obtaining parts of combination material and composites [97]. However, this technology has some disadvantages such as the necessary use of tiny sheets of material, different bonding processes for different types of materials and a non-high resolution of complex parts.

Only after 10 years that Kunieda published his work, the first report about LOM with ceramic materials appeared [98]. The authors of this work, Griffin et al., used sheets of alumina and zirconia for the manufacturing of solid parts with a high density after removing the binder and sintering at high temperatures.

After Griffin’s investigation, a large number of materials such as Al_2_O_3_ and SiC [99,100], binary composites like ZrO_2_/Al_2_O_3_, Si/SiC, TiC/Ni [101,102,103], or more complex systems, for example, LiO_2_–ZrO_2_–SiO_2_–Al_2_O_3_ (LZSA) glass–ceramic composite have been investigated for they use in LOM [104].

Despite the great effort made in the study of ceramics for LOM, in recent years no progress has been observed in this field. Thus, the application of this technique is restricted only for the manufacture of ceramic parts with simple geometry, and large sizes, which create a problem for its application in the production of advanced ceramic components that, are generally characterized by their complex geometry and much times for its miniature sizes.

#### 2.3.2. Fused Deposition Modeling (FDM)

FDM, also called fused deposition of ceramics (FDC), was first reported by Crump when he filed his own patent US 5121329 “Apparatus and method for creating three-dimensional objects” in 1989 [105]. Nowadays, this process is considered as the most usual AM technology in the world thanks to its accessibility, easy in use and low cost [106]. Different types of materials can be utilized in the FDM process, such as polymers, metals, and ceramic-or metal-filled polymers. The main concept of the FDM process is to create a part layer by layer by supplying a filament of semi-molten materials [107]. The feedstock is a thermoplastic polymer filament such as acrylonitrile butadiene styrene copolymers (ABS), polycarbonate (PC), Polyamide (PA) and polylactic acid (PLA) that is permanently providing to a nozzle which is heated at a temperature just above the filament melting point [108]. The semi-molted material is extruded through the moving controlled nozzle to form the desired pattern [13]. After the extrusion, the filament adheres to previously deposited layers and immediately cools allowing its solidification. When a layer is solidified with the desired pattern the part support is lowered a distance equal to the thickness of the new layer by an elevator system and a new cycle of extrusion and lowering is repeated until the final part is formed [14]. The schematic diagram of the FDM process is shown in Figure 5b [44]. Generally, FDM technology is widely used to manufacture parts with poor surface finish, low resolution, and mechanical properties. These low characteristics are commonly related to the influence of many factors such as material properties, air gap, printing orientation, raster angle, layer thickness, and raster width [109]. For this reason, the polymer parts obtained by FDM are often used as conceptual prototypes.

For the application of FDM in ceramic production, it is necessary to prepare filaments composed of binder thermoplastic polymers and tiny ceramic particles with a loading of about 60 vol%. In order to achieve a constant and stable flow of melt material, the ceramic particles should be well dispersed in the filaments, moreover, the viscosities of the melt filaments should be above 10 to 100 Pa·s [12]. Once the green body is obtained by FDM, the elimination of the binder polymer and the sintering are necessary to get the ceramic part. As Danforth reported in the first work about ceramic part fabrication by FDM in 1995 [110], the debinding step leads to the formation of pores that directly influence on the formation of defects and obtaining low densities. Despite this, thanks to the successful development of this technology and material science, the mechanical properties of parts obtained by FDM are now comparable with other processing routes [111]. This is why the application of FDM for ceramic production is successfully expanding into various fields as electronic components [112], biological parts [113], sensors [114], bioceramic scaffolds [115], and others [116,117].

#### 2.3.3. Direct Ink Writing (DIW)

Direct ink writing is a process related to the material extrusion group and it is also known as Robocasting [118], Direct Write Fabrication or Robot-Assisted Shape Deposition [119,120]. DIW was first reported by Cesarano and Calvert when they filed a patent at Sandia National Laboratories on October 28, 1997 [121]. DIW is an uncomplicated, responsive, and cheap process, appropriate for various materials, as follows: ceramics, metal alloys, polymers, and even edible materials [122]. Moreover, this is the most universal technique to produce the 3D prototypes [123], whose main goal is to make parts by extrusion of concentrated suspensions formulated of main material together with additives to get appropriate viscoelastic behavior [13]. The concept of this technique is very close to de FDM with the difference that the DIW process depends on the feedstock rheology behavior to maintain the shape of the printed part in the time [118]. The high viscosity (10^3^–10^6^ mPa s) pseudoplacticity performance of the feedstock are indispensable for this technique [123]. In this way, the pseudo-plastic feedstock is extruded through a moving controlled nozzle to form a desired two-dimensional pattern [13]. In DIW, the possibility of nozzle clogging is much lower than IJP. When a layer was printed the part support is lowered a distance equal to the thickness of the new layer. After that, a new layer is deposited on top of previously deposited forming a part thanks to a layer by layer procedure. As other technologies of ceramic production debinding and sintering post-processes are necessary to obtain a ceramic part without organics. Figure 5c demonstrates a schematic diagram of the DIW process.

This technology for ceramic materials, compared to SLA, is much faster and cheaper. The exclusive use of ceramic pastes with required viscoelastic behavior allows printing figures that can maintain their original form regardless of the loads generated by the newly deposited layers on them. Generally, the used pastes have a high loading of ceramic particles and the optimal content of additives. Thanks to this, it is possible to build parts with different configurations from complex porous scaffolds [25], to composite materials and solid monolithic parts [124,125]. Moreover, some researchers could prepare and use for printing filaments with different cross-sectional forms [126,127].

Thanks to the flexibility and simplicity of DIW, other scientists have been able to implement this technology for the fabrication of parts with periodic structures [128], for electrodes for lithium-ion (Li-ion) batteries [129] and, in recent years, the manufacture of bioceramic implants [130]. The last is the prominent application thanks to/as a result of the porosities that appear in the part after sintering. These porous structures are preferred in the manufacture of ceramic implants because they promote the growth of human body tissue in them [131]. Therefore, DIW fits very well for the manufacture of porous ceramic structures with periodic features, and when a little surface resolution is needed.

The aforementioned results show that DIW is a prominent technique for obtaining ceramic pieces with complex geometry, but with the great disadvantage that it is not possible to obtain highly dense pieces, which limits their application in the industry.

## 3. Additive Manufacturing for Graphene-Based Materials

As previously stated, in the last few years AM technologies have become so popular throughout the world that they have come to be applied in different fields of science and industry using different types of materials such as polymers, metals, ceramics, and composites. At the same time, in the last 20 years the development of materials science, specifically in the area of nanotechnology, has allowed the appearance, study, and development of interesting and perspective new materials, which are known as nanomaterials, for their application in the industry [32]. Micro- and nanomaterials of the same compound differ in that the latter can have exceptional and never-before-seen optical, electronic, and mechanical properties in comparison with the first. The great interest in nanomaterials is also because the properties of macro materials drastically change when nanomaterials are added to their structure [132]. Thanks to this, many ceramic-based composites that have a certain percentage of nanomaterials are converted into materials with improved mechanical properties. One of these promising nanomaterials is graphene [133,134]. Graphene is a revolutionary material that opens wide perspectives with its use, as an example, for increasing the flexural strength and fracture toughness of ceramic materials [135]. In the following sections, the characteristics and possible applications of graphene-based material, as well as the most popular additive technologies for them will be explained in more detail.

### 3.1. Graphene and Its Derivatives Materials

Graphene is a two-dimensional carbon allotropic form consisting of a single layer of sp2 hybridized atoms that are organized in a honeycomb lattice structure [136]. Graphene was discovered in 2004 by Andre Geim and Konstantin Novoselov, who worked at the University of Manchester [137]. This material demonstrates unique properties such as very high thermal conductivity (above 5000 W mK^−1^), high modulus of elasticity (1 TPa), large surface area (2630 m^2^/g), high electron mobility in room temperature (250.000 cm^2^/V s) and high tensile strength of 130 GPa [138]. Moreover, graphene shows high light transmittance, very high electrical conductivity, and complete impermeability to any gases, that make it a very promising material for a large number of multifunctional applications such as medicine [139], composite materials, electronics, light processing, supercapacitors, energy, strain sensors and others [138].

Different methods of obtaining graphene have been investigated, but the most used are chemical vapor deposition, epitaxial growth, the mechanical, oxidation–reduction method, and liquid phase and electrochemical exfoliation [138]. It should be noted that the widespread use of pristine graphene is limited mainly because it is hydrophobic [140]. The solution to this problem is the surface functionalization of graphene that is carried out by chemical modification [141]. Generally, chemical modification of graphene can be done in two ways: covalent, or non-covalent functionalization. Functionalization via non-covalent interactions creates a weak interaction of a π–π, van der Waals or electrostatic type between graphene and the target matter, while the covalent modification use the covalent bonding of oxygen-containing functional groups on the surface of graphene, forming carboxylic acid groups at the edges and epoxy and hydroxyl groups at the basal plane [142,143]. Usually, researchers around the world use processes based on the Hummers method, which are known as the modified Hummers method [144], for the covalent modification. The main idea of the Hummers method is the use of very strong oxidizing agents, such as concentrated sulfuric acid, nitric acid, and potassium permanganate, for the formation of oxygenated functional groups on the graphite, which is then mechanically exfoliated to obtain fine sheets of graphene with functional groups also on its surface. This material is called graphene oxide and it is hydrophilic that disperses easily in water [145].

The oxidation of graphene creates a large number of defects in its lattice structure that degrade the material properties, moreover, the functional groups in the GO surface make it electrically insulating [145]. Fortunately, a partial restore of graphene properties is possible thanks to a reduction process of GO that can be carried out mainly by chemical or thermal ways, although other less popular routes have also been used [146]. The idea of the reduction process is to eliminate the hydrophilic functional groups on GO surface to produce reduced graphene oxide (rGO) by the application of heat treatment or reducing agents in the thermal or in chemical way, respectively. 

Graphene and rGO differ from each other primarily by the presence of defects and some functional groups that remained in their structure after the reduction process. This fact turns rGO into a material with properties close to graphene and, at the same time, soluble in different media that allows it to be used in industry [147]. For instance, a large number of graphene-based composites have been created to improve the mechanical [148], thermal and electrical properties of polymer matrices used with applications in aerospace [149], electronics [150], and energy storage [145]. Furthermore, graphene-based composites with inorganic matrices, such as metals, ceramics, and composites have been developed [151,152]. 

### 3.2. Additive Technologies for Graphene-Based Materials

Like ceramic materials, graphene and its derivatives materials are also being studied to define their role and use in AM [153]. Since graphene oxide is hydrophilic, it is the most suitable material to be used as a precursor of graphene in additive technologies since this material can be easily dispersed in different solvents, and especially in water. Thus, the appropriate AM techniques to be used with graphene-based materials should belong to the categories vat photopolymerization, material jetting and material extrusion. In this review, only the AM techniques that are more typical for the production of graphene-based composites, in particular SLA, IJP, FDM, and DIW, are analyzed. Some of these methods are based on the use of polymers; and the introduction of graphene-based material into them allows the obtaining of polymer nanocomposites with improved properties, for example, barrier properties [154], optical properties [155], thermal properties [156], electrical properties [157,158] and mechanical properties [159,160].

The final properties of polymer nanocomposites crucially depend on the effectiveness of the nanoparticle dispersion process [161]. Thus, good nanofiller dispersion in the polymer will produce a maximum increase in the properties of the composite [162,163]. In many studies, the process of preparing composites has been taken into account to obtain a high homogeneity and dispersion of graphene-based materials within a polymer matrix [164]. The tactics implemented in different works can be summarized in three strategies [161]: 1–in situ intercalative polymerization. According to this technique, graphene oxide or graphene is first expanded in the liquid monomer, then an appropriate initiator is diffused and polymerization response use as the means of heat or radiation; 2–solution intercalation. In this case, the technology consists of three stages: dispersion of graphene or GO in a relevant solvent by sonication, the addition of polymer and removal of the solvent. Then, the graphene-based-solution is mixed with the polymer matrix, which is adsorbed onto the carbon sheets. Next, the carbon sheets sandwich the polymer to create a nanocomposite by removing the solvent, which is essential for the nanocomposites characteristics. The primary benefit of this technique is introducing low or even no polarity through the synthesis process; 3–melt intercalation. Graphene or GO and thermoplastic polymer mixing by mechanical means at high temperatures by extrusion or injection molding. This method is solvent-free. 

Of these three strategies the last two are the most widely used, while, as far as is known, data on the fabrication of graphene composites with the addition of polymer by in situ polymerization before being extruded into filaments for fused deposition modeling are scarce [165].

#### 3.2.1. Stereolithography (SLA)

In Section 2.2.1, the historical facts, basic principles, advantages and disadvantages of this technique were considered. It is necessary to take into consideration two essential issues for SLA-based composites: (1) rapid solidification of by light initiated polymerization, which requests a fast light-responsive composite resin system; (2) the reached low viscosity that enables for the dipping of the resin layer, and which defines a low graphene concentration and uniform filler distribution [166].

Over time, the SLA evolved and improved versions of this technique such as DLP, continuous liquid interface production (CLIP), projection microstereolithography (PμSL) and TPP [64,167,168,169] appeared. These modifications allow manufacturing parts with a higher resolution, shorter manufacturing time and low post-process requirements [170].

**Figure 6:**
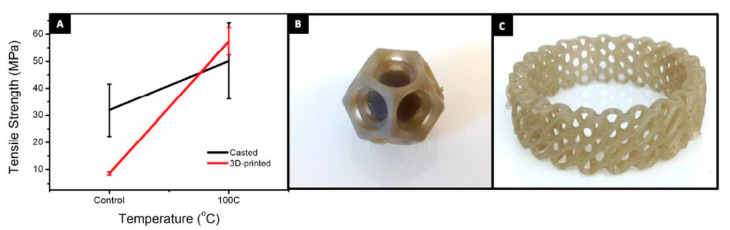
(**A**) Tensile strength comparison of casted and 3D-printed parts; SLA-printed complex-shaped graphene oxide (GO) nanocomposites: (**B**) nested dodecahedron and (**C**) diagrid ring. Reproduced from [171], with permission from American Chemical Society, 2017.

In 2015, Lin et al. reported the first manufacturing of GO reinforced complex architectures by mask projection-based Stereolithography (MPSL), also called digital light processing (DLP), with a good combination of strength and ductility [161]. First of all, the expected weight amount of GO nanosheets was sonicated in acetone and then they were dispersed in the polymer resin. This research demonstrated that tensile strength and elongation of printed composite parts with only 0.2 wt% GO increased by 62.2% and 12.8%, respectively. Moreover, this research group noticed a new fracture behavior of 3D printed truss architecture during compression testing. Later, in 2017 an experiment was carried out by Manapat et al. on the manufacture of high-strength nanocomposites by a typical SLA process. In this work, the authors used the GO metastable structure to improve the thermo-mechanical properties of a printed part that was then annealed at low temperatures. For this, composites with different GO contents (between 0 and 1 wt%) were prepared by dispersing the GO in acetone and then mixing the as prepared dispersion with a resin. The viscosities of the prepared GO resin varied between 0.6 and 1.6 Pa·s depending on the concentration of GO. Before, the as-obtained GO resins were used to manufacture the 3D parts with an axial resolution of 50 microns, which were then annealed at low temperatures (50 °C and 100 °C for 12 h). The best results were obtained after annealing at 100 °C for the 1 wt% GO nanocomposite that showed a very high increase of 673.6% on tensile strength compared with the casted material (Figure 6) [171].

**Figure 7:**
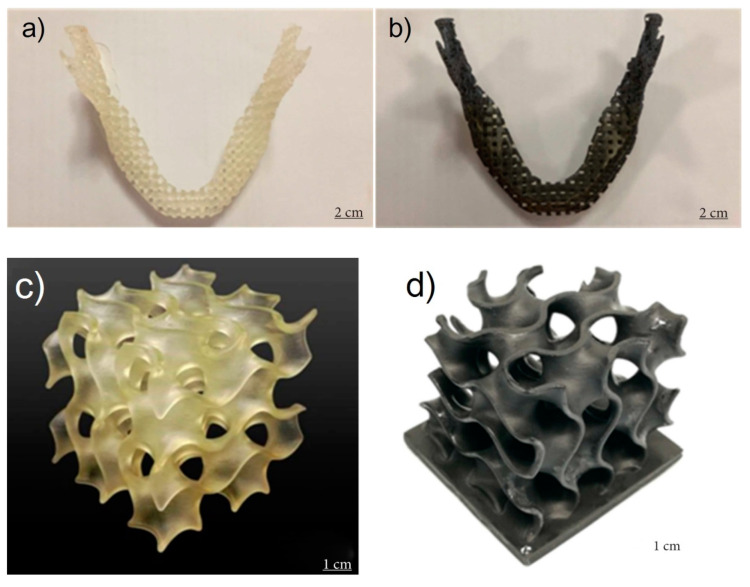
Pictures of (**a**,**c**) monolithic UV-cured resin and (**b**,**d**) graphene-reinforced nanocomposite jawbone with a square architecture and gyroid scaffold for bone tissue engineering, respectively. Reproduced from [172], with permission from Zuying Feng et al., 2019.

The previous work result shows that SLA is a good candidate to be used in the rapid manufacturing of parts based on graphene reinforced composites that can be used in different applications, such as in the biomedicine field. For example, in the work published in 2018, a graphene-reinforced composite for bone structure scaffolds was reported. Here, Feng et al. invented a biodegradable UV-cured resin by SLA to create the personalized complex structure for bone tissue scaffolds, which have been reinforced by the filling of graphene layers [172]. The composite consists of an easily accessible polyurethane resin, trimethylolpropane trimethacrylate (TEGDMA) and phenylbis (2, 4, 6-trimethylbenzoyl)-phosphine oxide (Irgacure 819) as an oligomer, a reactive diluent and a photoinitiator, respectively. The obtained resin had suitable viscosities for SLA in the order of 847 Pa·s (at 25 °C) and 500 Pa·s (at 30 °C). Thanks to the inclusion and the good dispersion of graphene fillers, the manufactured parts by SLA had improved their mechanical performance compared with traditional direct casting techniques. Thus, the tensile strength of the printed part rose to 68 MPa from 42 MPa that is the value for the same material but produced by direct casting. Other improvements are also been registered in the flexural strength (115 MPa) and flexural modulus (5.8 GPa). Consequently, these results show that this graphene-reinforced resin has a great ability to produce biotissue compared to the conventional mold-based step by step techniques that have a considerably low cost. Figure 7 shows the images of jawbones, and gyroid scaffolds of pure UV-cured resin and graphene-reinforced nanocomposite manufactured in this work.

**Figure 8:**
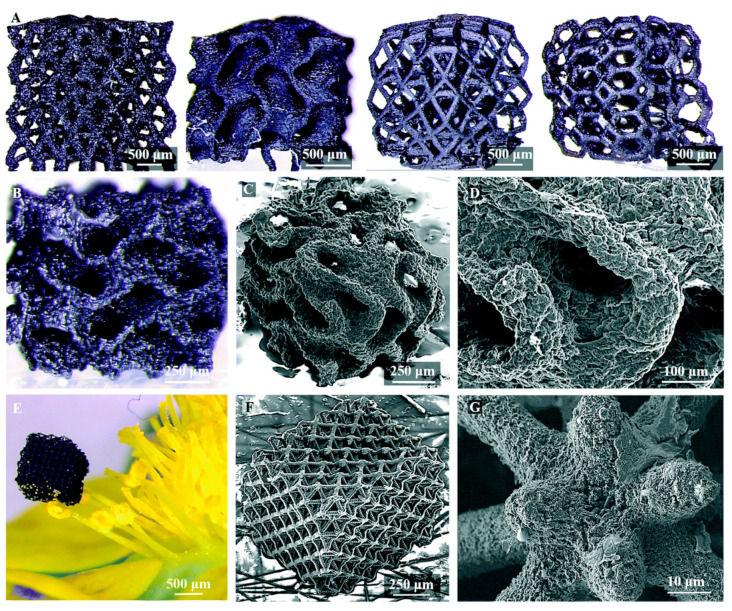
(**A**) Four “Green” MAG parts of differing unit-cell structures before pyrolysis from left to right: octet-truss, gyroid, cubo-octahedron, and Kelvin foam; (**B**) optical image of pyrolyzed gyroid; (**C**) SEM image of pyrolyzed gyroid with intricate overhang structures (**D**) zoomed image of pyrolyzed gyroid in (**C**); (**E**) optical image of pyrolyzed MAG octet-truss, of a different design than shown in (**A**) supported by a single strawberry blossom filament; (**F**) SEM image of pyrolyzed octet-truss MAG in (**E**); (**G**) zoomed image of octet-truss in (**E**) showing the very high 10 micron resolution achievable in our process. Reproduced from [173], with permission from the Royal Society of Chemistry, 2018.

A short time ago, Hensleigh et al. investigated the manufacturing of complex micro-architected graphene aerogels by using an “XGO” resin. In this study was demonstrated that graphene containing resin could be precisely designed to any complex shape with 3D spatial characteristic sizes of ~10 microns (Figure 8G, [173]) by light, which is a much higher resolution compared with other works where the obtained feature sizes were on the order of 100 μm.

In the current state of the art, other graphene-based 3D parts obtained by SLA improved versions as DLP [174], TPP and others can be found [170,175].

#### 3.2.2. Inkjet Printing (IJP)

This technology was considered in Section 2.2.4 of this paper. For the accurate fabrication in this technique, it is necessary to make spherical droplets that can be obtained by an optimal choice of jetting characteristics, as follows: voltage, frequency, and viscosity waveform [123]. Besides, the used feedstocks commonly are low-viscosity suspensions with the desired flow behavior, which is determined by their viscosity, shear elastic and yield stress points and surface forces that form a layer on the surface of suspension [176]. Moreover, the nozzle diameter *α* (μm) and density *ρ* (g/cm^3^), surface tension *γ* (mN/m), viscosity *η* (mPa·s) of the ink directly influence on the drop formation [176]; and these ink characteristics must be taken into account during the preparation of the graphene-based feedstock [177,178,179]. One of the methods for ink preparation is the liquid phase exfoliation [180], in which graphite is first dispersed in a solvent and then exfoliated by sonication. Unfortunately, solvents that could give a better exfoliation result, especially N-methyl pyrrolidone (NMP) and dimethylformamide (DMF), are highly toxic. For this reason, studies are constantly carried out to find a replacement for these toxic materials [180,181,182]. Despite the efforts that were made to obtain such materials, the dispersity of graphene suspensions remained poor. A very practical way to stabilize the inks is to use GO or rGO together with surfactants that improve the dispersity of sheets [180,183,184], thereby it reduces the probability of agglomerate formation in graphene-based inks.

Different studies on IJP with graphene-based inks have been carried out, mainly for applications in electronic, bioelectronic, and energy storage [185,186,187,188,189,190]. For example, Li et al. reported an easy IJP method for the fabrication of micro-supercapacitors (MSCs) based on graphene and printed on various substrates. The authors prepared an ink based on high-performance graphene with dimethylformamide (DMF) that had a level of 2.3 mg/mL of graphene sheets and a sustained trend of more than half a year. This suspension was used for the fabrication of very thin films (with a depth up to ~0.7 μm) which are used as electrodes and current collectors. The fully printed graphene-based MSCs demonstrated an extremely high capacitance of about 0.7 mF/cm^2^, which considerably exceeds the maximum value reached (~0.1 mF/cm^2^) in printed graphene-based MSCs [191,192] before this study. Moreover, the authors demonstrated that this approach enabled the multi-scaled production of MSCs and outstanding connection in parallel and/or in series [165], for instance, over 100 devices were connected to create large-scale MSC arrays as power banks on Kapton and silicon wafers. Free from extra protection or encapsulation, the MSC arrays can save their efficiency, for a 12 V charging, even eight months after fabrication.

**Figure 9:**
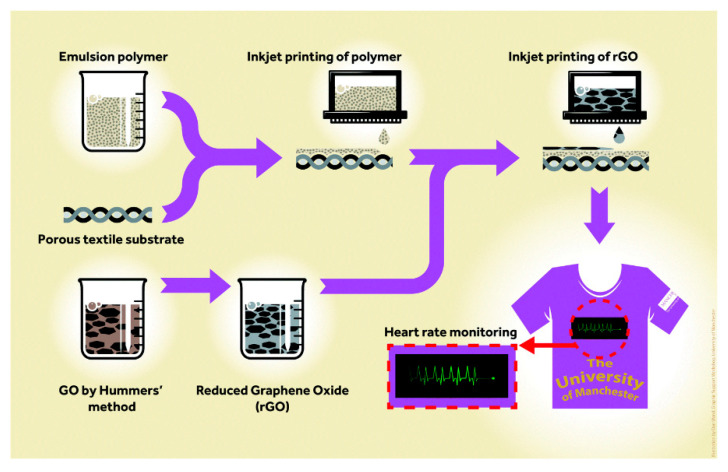
Diagram of the IJP process of graphene-based inks for e-textile manufacturing. Reproduced from [193], with permission from the Royal Society of Chemistry, 2017.

**Figure 10:**
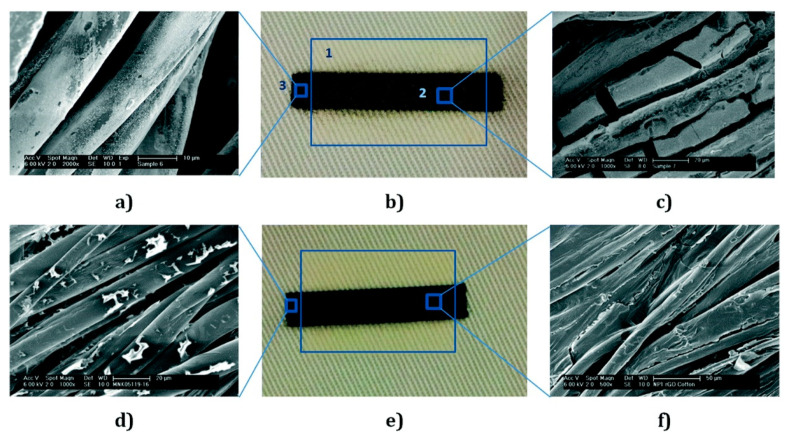
The different conductive paths, which were IJP on the untreated and treated areas of the cotton fabric with NP1. (**a**,**c**) show the SEM images of the untreated cotton fabric coated with 6 layers of IJP silver ink (×2000), and the IJP silver conductive path (6 layers) onto treated cotton fabric with 12 layers of NP1 (×1000), respectively. (**b**,**e**) show 3 different areas of the cotton fabric for silver and rGO ink, respectively: (1) area printed with 12 layers of NP1; (2) 6 layers of IJP silver (or rGO) conductive path onto NP1; and (3) untreated cotton fabric coated with 6 layers of IJP silver (or rGO) ink. (**d**,**f**) show the SEM images of the untreated cotton fabric coated with 6 layers of IJP rGO ink (×1000), and the IJP rGO conductive path (6 layers) onto treated cotton fabric with 12 layers of NP1 (×500), respectively. Reproduced from [193], with permission from the Royal Society of Chemistry, 2017.

Karim et al. first reported a pre-treatment of a textile surface for inkjet printing using organic nanoparticle-based ink [193]. This treatment permits the printing by IJP of any wearable e-textiles based on graphene. In this study, the authors developed the printing process on porous and rough textile material of a conductive path, because it is the main problem related to inkjet printing of conductive inks on textiles. Figure 9 displays a diagram of the IJP process of graphene-based inks for e-textile manufacturing. The textile surfaces were pre-treated with hydroxyl functionalized cross-linked styrene/divinylbenzene nanoparticles (NP1); on the other hand, the aqueous ink of reduced GO was obtained utilizing L-ascorbic acid in polyvinyl alcohol (PVA), which act as a non-toxic reducing agent. Later, a continuous conductive electrical path of water-based rGO inks onto the pre-treated coating was printed by inkjet printing. The results of this study show that this approach reduces the sheet resistance of graphene-based printed e-textiles by three orders of magnitude from 1.09 × 10^6^ Ω/sq to 2.14 × 10^3^ Ω/sq compared with untreated textiles. Here, the pre-treated surface acts as a receptor of the aqueous ink of reduced GO, which is after that dried at 100 °C; thus the chance of harm to the heat-sensitive fabrics is reduced. In this way, the IJP process of aqueous ink of bio-compatible reduced GO provides opportunities for the fabrication of next-generation e-textiles for military, healthcare, and sports applications. In Figure 10 are shown the different conductive paths, which were IJP on the untreated and treated areas of the fabric with NP1. Figure 10a is an SEM image (×2000) of the untreated cotton fabric coated with 6 layers of IJP silver ink. In Figure 10b are appreciated thee different areas of the cotton fabric: (1) area printed with 12 layers of NP1; (2) 6 layers of IJP silver conductive path onto NP1; and (3) untreated cotton fabric coated with 6 layers of IJP silver ink. Moreover, in Figure 10c is possible to see an SEM image (×1000) of the IJP silver conductive path (six layers) onto treated cotton fabric with 12 layers of NP1. On the other hand, Figure 10d–f show the image of the untreated cotton fabric coated with 6 layers of IJP rGO ink (×1000), the IJP conductive rGO paths on the untreated and treated areas of the fabric with NP1, and SEM image (×500) of IJP rGO conductive path (six layers) onto treated cotton fabric with 12 layers of NP1.

**Figure 11:**
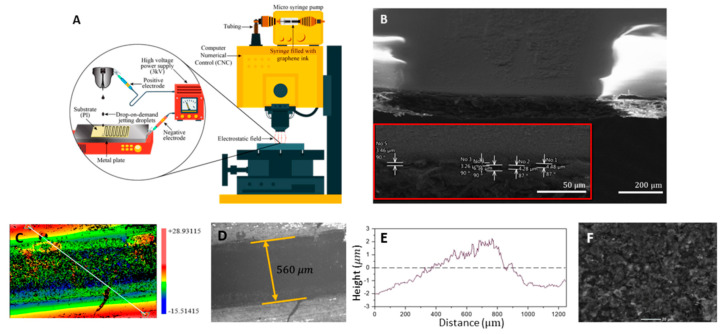
(**A**) DOD printer for the printing of graphene suspension; (**B**) SEM image of printed pattern cross-sectional view; (**C**) image of printed graphene suspension a contour in which the average height was measured through the white line. (**D**) Sample print section (20× magnification). (**E**) height vs. distance of the sample line in (**C**). (**F**) SEM image of the printed graphene suspension top view after annealing. Reproduced from [187], with permission from the authors, 2020.

Recently, Asli et al. proposed a method for high-efficiency preparation of graphene-based aqueous suspension for electrohydrodynamic DOD printing of conductive patterns [187]. Here, an exfoliation process of graphite in water that is easy and scalable to high yield was suggested for the first time. This approach can prepare high-quality graphene thanks to the combination of the sheer force of continuous low-speed wet ball milling process together with Bovine Serum Albomine (BSA) that acts as an exfoliating agent. In this way, the exfoliation of graphite particles to 2–3 layers graphene sheets on average can be achieved. The as-obtained graphene suspensions possessed a concentration of 5.1 mg/mL and it remained stable for weeks. This stable graphene dispersion is preferred for the printing process, as it dramatically reduces the probability of nozzle clogging. Then, the prepared ink was printed on a flexible substrate (Polyimide) with the controlled resolution by the use of an electrostatic field in the drop-on-demand printer. After printing, thermal annealing is essential for the improvement of the conductivity of the printed few-layer graphene. On the one hand, it is necessary for the BSA combustion, which is fundamental for the pattern stability when they are in contact with water; and on the other hand, it diminishes the defects between graphene and substrate and graphene sheets that improve the order of the surface morphology. Thus, the post-processing was carried out in different durations (from 10 to 120 min), and temperatures (from 50 °C to 280 °C). The results demonstrated that no-annealed layer had a sheet resistance of 133 Ω/sq, while annealed samples reduced the sheet resistance to 36.75 Ω/sq in a standard oven at 280 °C with a duration of 30 min. After annealing, the conductive layers did not lose their adhesion to Polyimide they were in contact with water. This approach can be used for innovative biosensor applications, as well as other applications in printable and flexible electronics. Figure 11, [187], displays SEM and optical analysis of printed patterns.

#### 3.2.3. Fused Deposition Modeling (FDM)

Thanks to the facility to add different phases in the thermoplastic matrix, this technology can be used for the production of parts with a wide type of composite materials [194]. As an example, the inclusion of electrically conductive carbon allotropic forms like graphene, graphite, carbon nanotubes, and carbon black into the filament of the printed parts can demonstrate an improvement in electrical properties and, as in the case of graphene and CNT, in mechanical properties [153,195,196].

The first study about the possibility of using graphene-based compounds in FDM to manufacture parts was reported by Wei et al. [197]. In this work, the solution intercalation strategy was used. The authors utilized N-Methylpyrolidone (NMP) as a solvent, to get good dispersion of ABS and GO. After the GO was reduced some quantities of rGO/ABS powder formed and precipitated from solution. Next, this powder was used for the preparation by extrusion of a filament that would later be used for the manufacture of parts by FDM. The maximum content of graphene in the manufactured filaments was 7.4 wt%.

In recent years, different studies have been carried out on the application of graphene-based composites in FDM to be applied in different areas, as well as biomaterial scaffolds [198], electrochemical energy storage architectures [199,200], and flexible circuits [201].

**Figure 12:**
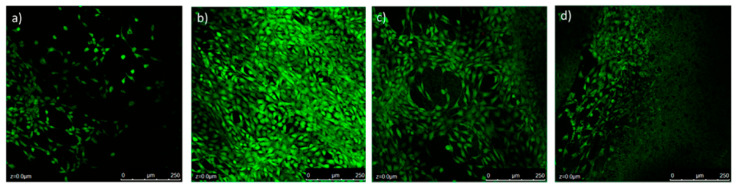
96 h cell culture results of NIH3T3 cells on 3D printed TPU/PLA with different GO loadings: (**a**) 0 wt% GO, (**b**) 0.5 wt% GO, (**c**) 2 wt% GO, (**d**) 5 wt% GO. Green color indicates live cells, whereas red color indicates dead cells. Adapted from [198], with permission from American Chemical Society, 2017.

For example, in 2017 Chen et al. fabricated scaffolds for tissue engineering by application of thermoplastic polyurethane (TPU)/PLA/GO nanocomposites and explored their biocompatibility. These nanocomposites were obtained by a solution intercalation strategy and then the as obtained mixtures were precipitated in alcohol to obtain the precipitates. Next, the precipitates were dried and extruded for the fabrication of nanocomposite filament. Diverse amounts of GO (0.5, 2, and 5 wt%) were mixed with polymer with a fixed ratio of TPU/PLA equal to 7:3. Thereby, a monolayer of the as-prepared composite was printed on a glass substrate; then the live/dead viability/cytotoxicity assay using NIH3T3 mouse embryonic fibroblast cells was carry on. According to research, in the structure of the obtained 3D printed composites only live cells were observed, i.e., all scaffolds provided cell growth. The maximum density of cells was reached in the scaffold with 0.5 wt% GO, Figure 12b [198].

**Figure 13:**
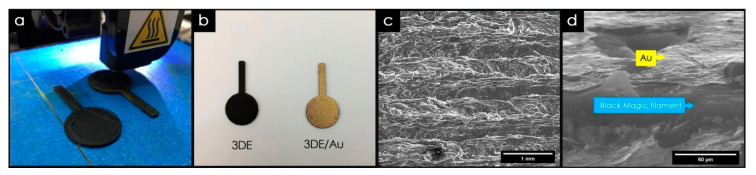
Physiochemical characterization. (**a**) Optical image of the 3D printing process, (**b**) 3D printed electrode used throughout the study. (**c**) FESEM image of 3DE/Au electrode, and (**d**) corresponding magnified cross-sectional area. Reproduced from [202], with permission from Springer Nature, 2018.

**Figure 14:**
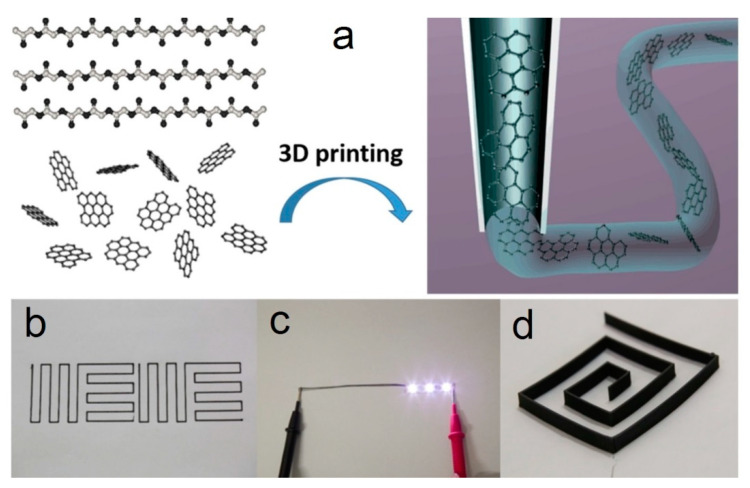
(**a**) Simplified schematics depicting the process of graphene-based 3D printing by FDM; (**b**) two units of 3D printed paper-based flexible circuits pattern; (**c**) LED circuit with a bunch of 3D printed filaments; (**d**) 3D printed flexible circuits. Reproduced from [201], with permission from Elsevier, 2016.

Foo et al. developed a method for producing 3D printed electrode (3DEs) and its novel applications in electronic devices. Here, the 3Des were created by employing a commercial graphene-based conductive filament that was purchased from Black Magic. Besides, a coat of gold was deposited on the surface of the 3DEs for the complete fabrication of the electrode, which was named 3DE/Au, Figure 13 [202]. The 3DE/Au was used as the current collector and working electrode for a solid-state supercapacitor with a multilayered structure. Before the assembly of the supercapacitor, a layer of polypyrrole/reduced graphene oxide (Ppy/rGO) nanocomposites were deposited on the 3DE/Au face in-situ by means of electrochemical polymerization technique. The assembled supercapacitor showed appropriate capacitance behavior with a specific capacitance of 98.37 F/g. These 3DEs were fabricated into a photoelectrochemical sensing platform that had a photocurrent response at ~724.1 μA and a lower detection limit (0.05 μM) compared to the indium (ITO)-or fluorine-doped tin oxide (FTO) glass electrode. Zhang et al. manufacture by FDM highly conductive graphene flexible circuits. In this study, the authors prepared high conductive graphene with a conductivity above 600 S/cm by a two-step in-situ reduced method. The first step was a chemical reduction by 4-iodoaniline, while the next stage included a thermal reduction in the Ar atmosphere at 1050 °C for 1 h. The conductive (4.76 S/cm) filaments of PLA-rGO composite were finally fabricated by homogenously mixing rGO (6 wt%) using melt intercalation. SEM images showed that graphene was well dispersed in the PLA substrate. The authors demonstrated that the orientation of r-GO fillers takes place during the extrusion process, and this effect contributes to the increase in the conductivity of the filaments. In addition, the 3D flexible circuits exhibit good bonding force between layers, indicating that the 3D structure can maintain the same good mechanical property in both the axial direction and transverse direction. Besides, the manufactured 2D flexible circuits on paper and polyimide (PI) substrates showed a great bonding force between the composite circuits and both substrates, Figure 14 [201].

#### 3.2.4. Direct Ink Writing (DIW)

Direct ink writing is an AM technique based on the extrusion and deposition of a pseudo-plastic material (paste), which can maintain the shape of the extruded filament and the printed part in the time after extrusion. Previously, we had already considered this technique at the Section 2.3.3 of the present work.

Among the all AM technologies, direct ink writing is one of the most broadly used for the manufacturing of 3D parts from a graphene-based feedstock tanks to the combination of the great possibilities of DIW with the unique properties of graphene that has shown noteworthy printing capabilities and unique viscoelastic properties [203].

**Figure 15:**
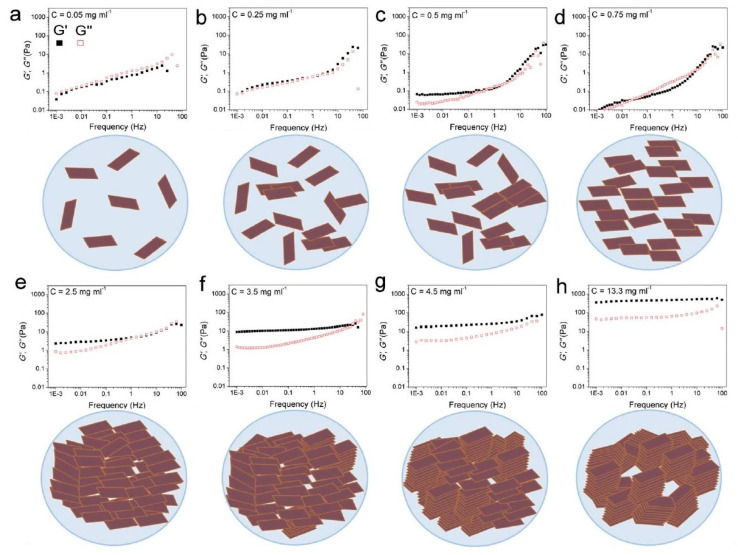
Storage (filled squares) and loss moduli (open squares) of graphene oxide suspensions and the schematic illustrations of the liquid crystal (LC) phase changes upon the increasing concentration of the graphene oxide suspensions. (**a**) At extremely low concentration. (**b**,**c**) Some nematic ordering begins to appear when the concentration increases to 0.25 mg/mL. (**d**) In the dispersion single-phase nematic LC starts to form. (**e**) The increase of the nematic phase packing is higher with the increase of the GO concentration. (**f**) Some regions of GO exhibit orientation in the nematic phase. (**g**,**h**) Smaller monodomains are formed associated with an exceptional increase in elastic modulus. Adapted from [204], with permission from The Royal Society of Chemistry, 2014.

**Figure 16:**
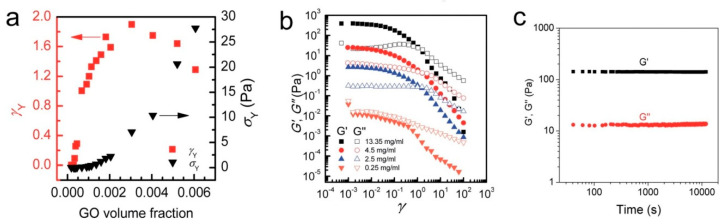
Fingerprints of the rheological characteristic of LC GO dispersions. (**a**) Yied stress (*σ*Y) and yield strain (*γ*Y) versus GO volume fraction. (**b**) Storage and loss moduli of GO suspensions versus strains (frequency of 0.01 Hz). (**c**) No aging after shear fluidization can be observed. Adapted from [204], with permission from The Royal Society of Chemistry, 2014.

Naficy et al. reported that graphene-based pastes with concentration up to 13.3 mg/mL are suitable for DIW. In Figure 15 the storage (filled squares) and loss moduli (open squares) of graphene oxide suspensions and the schematic illustrations of the liquid crystal (LC) phase changes upon the increasing concentration of the graphene oxide suspensions are showed. In this work, the GO ability to dissipate stress through heat at 13.3 mg/mL was measured and its value was found to be in the range of 350 to 490 Pa. These values considerably exceed the calculated elastic modulus value (~60 Pa) of a single-wall nanotube (SWNT) suspension with the same concentration. The rheological behavior of LC GO dispersions are is shown in Figure 16 [204].

**Figure 17:**
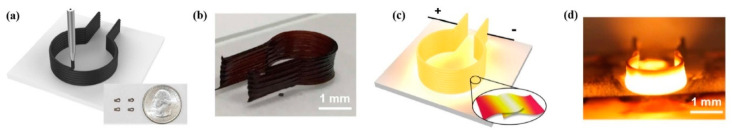
Schematic demonstration of the 3D printable heater. (**a**) 3D printing of RGO heater. The inset has 4 heaters shown with a size of 1.5 mm. (**b**) The image of the as-printed 3D heater. (**c**) The RGO heater achieves temperatures above 3000 K when a driving current is applied. (**d**) image of 3D printed heater at high temperature. Adapted from [28], with permission from American Chemical Society, 2016.

In other work, Yao et al. fabricated by DIW high-temperature and high rate heaters by using an aqueous paste with a high concentration of GO (80 mg/mL) [28]. The apparent viscosity of the paste falls in a range of 10^2^ and 10^3^ Pa·s (at a shear rate of 1 s^−1^), while the storage modulus (G′) shows a constant value at 10^4^ Pa, while the shear stress was in the order of 10^−1^ Pa and 10^2^ Pa. These values are appropriate for the printing by DIW where parts are formed by layer-by-layer stacked architectures in order to have good shape retention. In the experiments, the prepared heaters generated high temperatures up to 3000 K in a monitored form, while the temperature ramping response was fast and the heating rate was up to ~20,000 K/s. Moreover, 3D heaters also exhibited high working stability at high temperatures including a gradual change in temperature in the ambient temperature range and 2000 K over 2000 cycles. Figure 17 shows schematic illustrations and pictures of the 3D printable heater.

In 2014, were first reported 3D-printed nanostructures composed entirely of graphene by Kim et al. [205]. It was stated that a meniscus-guided growth technique was adopted to write free-standing reduced graphene oxide (rGO) nanowires without any supporting materials by a micropipette. Due to the very small open diameter (1–2 μm) of the micropipette, the water would evaporate very fast which led to the solidifying of the GO suspension by pulling the micropipette. Thanks to the high control of moving various freestanding graphene structures could be printed with 100 nm resolution, ranging from straight wires, bridges, suspended junctions to woven structures [166].

Diverse studies about DIW of graphene-based materials with diverse applications, like as scaffold [203], Li-ion battery [122], and supercapacitor had been reported too [206]. For example, Jakus et al. fabricated multifunctional microsystems by 3D printable graphene (3DG) composite for electronic and biomedical applications [207]. In this study, graphene powder and polylactide-co-glycolide (PLG) were mixed in dichloromethane (DCM). During extrusion, the fast evaporation of DCM provided a self-supporting filament that would not be changed after deposition. The composites had a maximum graphene loading of 60 vol%. Additionally, the authors demonstrated that during the extrusion a flakes orientation occurs along with the filament microstructure. Graphene particles stacked within the filament but aligned in the exterior of the filament.

Fu et al. produced Li_4_Ti_5_O_12_ (LTO)/GO and LiFePO_4_ (LFP)/GO composites for the AM of Li-ion battery by DIW [122]. The composites showed a high electrical conductivity after the thermal annealing of GO. Composite pastes were obtained by adding an LFP or LTO to GO suspension (80 mg/mL) with a mass ratio of 7:3, in which only water was de solvent. The storage modulus (G′) of both pastes was in the region of 10^4^ to 10^5^ Pa during the plateau region, which indicated a stiffer ink with a solid-like response. On the other hand, elastic limit values for the two composite pastes were 10^3^ Pa. These two high values are necessary for the paste application in DIW. After the obtaining of electrodes were freeze-dried and then thermally annealed in Ar/H_2_ gas. The initial charge and discharge capacities of LFP/rGO electrode, at a specific current of 10 mA/g, were 168 and 164 mAh/g, respectively, that are very close values to the theoretical capacity of LFP (170 mAh/g); while LTO/rGO electrode showed values of 184 and 185 mAh/g, respectively, that are higher than the theoretical capacity of LTO (175 mAh/g). On the other hand, the fabricated battery demonstrated initial charge and discharge capacities of 117 and 91 mAh/g at a specific current of 50 mA/g.

Liu et al. studied the preparation of graphene oxide/polyaniline (PANi) feedstock for flexible micro-supercapacitors (fMSCs) [206]. The authors made composites with aligned PANi nanorods, which were vertically grown on the two surfaces of the GO sheets. For this, the vertical nanorods were fixed on the graphene oxide surfaces by an interfacial polymerization approach; and then, a clearly defined nanostructure of GO/PANi composite was obtained. Next, the conductive poly(3,4-ethylenedioxythiophene):poly(styrenesulfonate) (PEDOT:PSS) was utilized as a dispersing agent to achieve highly dispersed aqueous GO/PANi-PEDOT:PSS (GO/PA-PE) feedstock with the appropriate rheological behavior for printing by extrusion. Here, PANi nanorods were wrapped by PEDOT:PSS changing their morphology giving them a much smoother surface than the started sharp protrusions. The PEDOT:PSS application provides the formation of a material with high electrical conductivity and permits the complete utilization of inner surface capacitance, in addition to improving its printability properties. The printed flexible micro-supercapacitors from GO/PA-PE showed high volumetric capacitance (19.2 F/cm^3^ at 5 mV/s) and areal capacitance (153.6 mF/cm^2^ at 5 mV/s) values that were greater than the literature values. Moreover, the authors demonstrated that by fabricating asymmetric fMSCs using the GO/PANi as the positive electrode and a graphene-based negative electrode, the voltage window can be widened from 0.8 to 1.2 V and improvements can be achieved in energy density (from 3.36 to 4.83 mWh/cm^3^), power density (from 9.82 to 25.3 W/cm^3^), and cycling stability (from 75% to 100% capacitance retention over 5000 cycles) compared with the symmetric counterpart.

## 4. Direct Ink Writing Technology of Graphene-Based Ceramic Pastes

DIW is among the most commonly used AM technique for the production of 3D parts from a graphene-based paste. For obtaining a part with good properties by DIW a high graphene content paste and with a suitable raw material is necessary [203]. Besides, a high colloid volume fraction in the paste will minimize the drying-induced shrinkage after printing. Very often, the use of additives (binders, viscosifiers, among others) is needed to provide a good dispersion of the graphene-based materials and obtain a paste with appropriate viscoelastic properties. In DIW, the paste viscosity for printing, which is related to the loss (i.e., viscous) modulus (G″), should be in the order of 10^3^–10^6^ mPa·s, which are very high values. On the other hand, the storage (i.e., elastic) modulus (G′) is associated with the paste elastic property thus, high values of G′ are required, because the higher G′ the stiffer is the paste with a solid-like response [165]. The yield stress and storage modulus G′ will be restored during ink exit from the nozzle, i.e., will remain their shape and dimension.

In the state of the art of graphene-based/ceramic 3D printed composites by DIW, diverse works with different applications as conductive ceramic nanocomposites [208,209], energy storage/conversion systems, high-temperature filters, and others, can be found.

**Figure 18:**
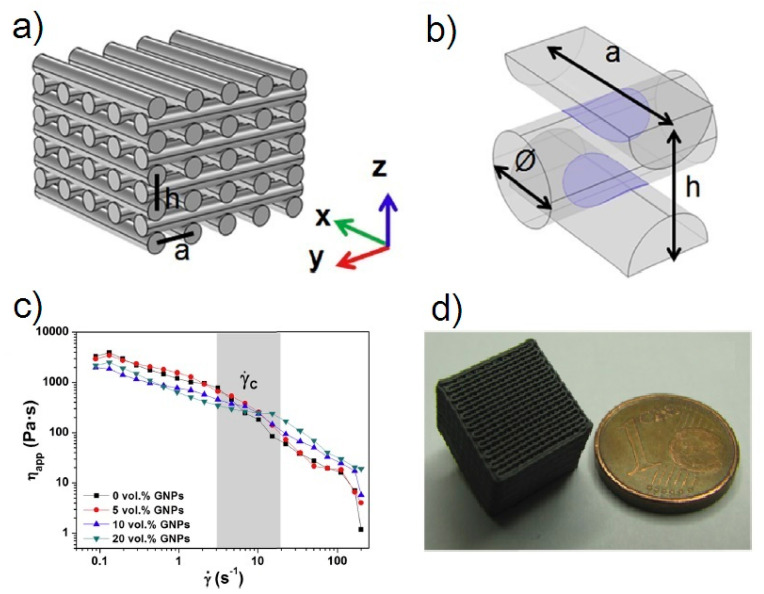
(**a**) Patterned structure used for scaffolds designing and (**b**) scheme of the contact area between two orthogonal rods, where *h*, *a*, and *Ø* correspond to the distance between two equivalent layers in the z direction, the distance between two adjacent rods, and the rod diameter, respectively. (**c**) Apparent viscosity as a function of the shear rate for the GNPs/SiC pastes formulated with 0, 5, 10 and 20 vol% GNPs in the powder compositions. (**d**) View of a 10 vol% GNPs/SiC sintered scaffold. Reproduced from [208], with permission from Elsevier, 2016.

Roman-Manso et al. first reported the study of 3D architected graphene/ceramic composites obtained by DIW. These composites are applied in energy storage/conversion systems, high-temperature filters, or as catalyst supports, gas sensors, and acoustic metamaterials. These 3D objects were printed starting from a paste containing homogeneous mixtures of SiC ceramic powders and up to 20 vol% of graphene nanoplatelets (GNPs), and then, these objects were consolidated by Spark Plasma Sintering (SPS), Figure 18 [208]. The paste was prepared as follows: three powder compositions were formulated with diverse GNPs contents (5, 10 and 20 vol%). The ceramic powder was mainly composed of b-SiC and using Al_2_O_3_ and add Y_2_O_3_ as sintering aids, and holding the SiC:Al_2_O_3_:Y_2_O_3_ formulation constant at a 93:2:5 (wt%) ratio for all the compositions. To obtain a homogeneous powder composition, the aforementioned components were mixed in an attrition mill with alumina balls in an isopropyl alcohol media. At the same time, a stable dispersion of GNPs in isopropanol was prepared by sonication. Next, the ceramic composite and the GNPs dispersion were mixed and, finally, stirred and sonicated. Subsequently, the solvent was removed in a rotary evaporator, and the mixture was dried at 120 °C and sieved through a 63 µm mesh. With aim of preparing the pastes, well-dispersed suspensions of the as-obtained dried blend in an aqueous polymer solution of polyethylenimine (PEI), methylcellulose (MC) and ammonium polyacrylate (APA) were obtained in a planetary centrifugal mixer. In these suspensions, PEI, MC, and APA acted as a dispersant, viscosifying agent and flocculant, respectively. The aqueous polymer composition for pastes with contents up to 10 vol%. of GNPs was (4 wt% of PEI, 5 wt% of MC and 0.3 wt% of APA); while the paste with 20 vol%. a slightly higher of the PEI concentration (5 wt%) to obtain the required pseudoplastic properties. Note that the solids concentrations in the pastes were in the range of 69–71 wt% (42–44 vol%) in all cases. Next, 3D architected composites were manufactured using a DIW printer. After printing, the parts were heated up to 415 °C to burn out the organics and, then, the as-printed parts were sintered in an SPS furnace at 1800 °C and an Argon atmosphere. Sintered composites showed high porosity, ranging from 1.6 to 0.9 g/cm^3^ for corresponding GNPs contents of 0 to 20 vol%, as compared with theoretical values of the bulk compositions 3.28 g/cm^3^ and 3.03 g/cm^3^ for the monolithic SiC and for the 20 vol% GNPs composites, respectively. Besides, the electrical conductivity of the scaffolds demonstrates some anisotropy with the architecture character and grows with the GNPs volume fraction. It was stated that, under such an approach, the values of up to 611 and 273 S/m for the longitudinal and transverse orientations, respectively, of the structures relative to the extruded cylinders were obtained. This anisotropy was determined by the design of the structure and also by the strong preferential orientation of the GNP within the rod during the printing process.

**Figure 19:**
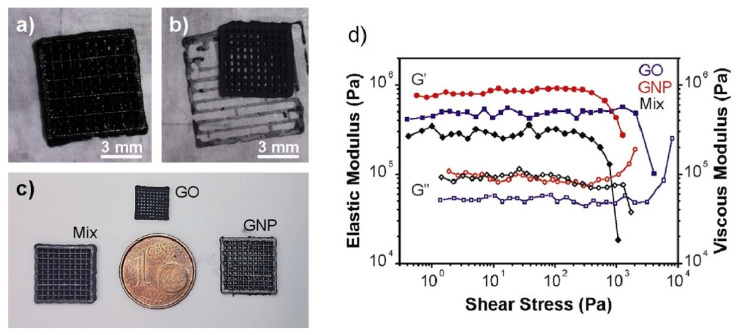
(**a**) 3D printed GO structure, (**b**) “a” dried 24 h in air; (**c**) Comparison of structures obtained after treatment at 1200 °C from GNP, GO and mix compositions; (**d**) Storage (G′) and loss (G″) moduli versus shear stress for the three inks: GO, GNP and mix. Reproduced from [211], with permission from Elsevier, 2019.

Tubio et al. proposed a scalable fabrication of rGO/Al_2_O_3_ composites with complex mesoscale architecture by DIW for their use in diverse applications [210]. The paste production involved three basic steps: dispersion, mixing, and gelation. In the first step, an aqueous Al_2_O_3_ colloidal suspension with diverse graphene oxide concentration (0.5, 1 and 5 wt%) was prepared in a planetary mixer. Then, the concentration of the as as-prepared suspension was increased by water evaporation at room temperature and mixed again several times. Subsequently, hydroxypropyl methylcellulose (HPMC) was added to increase the viscosity followed by other mixed steps. Next, polyethylenimine (PEI) was added to facilitate the gelation followed by other mixed steps. The rheological tests under steady and dynamic shear conditions were carried out to investigate the printability of as-prepared pastes. The data results showed two important effects: all pastes have shear-thinning (i.e., pseudoplastic) behavior, and the GO concentration influence on the viscosity data in the studied shear-rate range. Moreover, the highest apparent viscosity was found in the paste with graphene oxide concentration of 5 wt% and this paste showed a storage modulus (G′) ~1 × 10^6^ Pa, while the shear yield stress raised to 220 Pa from 20 Pa for paste with 0 wt% GO. Therefore, paste with 5 wt% GO content was used for the fabrication of GO/Al_2_O_3_ composites with complex mesoscale architecture by DIW. The rGO-Al_2_O_3_ composites were sintered in a protective atmosphere (N_2_) at 1600 °C. In another work, Moyano et al. proposed a new formulation of graphene-based pastes for producing self-supported 3D architectures by DIW. Here, the authors showed that is possible to obtain graphene-based pastes from just a single surfactant to achieve a suitable high elastic modulus and a shear-thinning behavior at rest. At the same time, the whole paste produce process is simple and scalable. Three aqueous graphene-based pastes were created by mixing GO, GNP and their mixture (GNP (92.7 wt%) and GO (7.3 wt%)) with an aqueous solution (30 wt% concentration) of Poloxamer 407, a triblock copolymer that contains 70 wt% of PEO units. Pastes with 30 wt% solution of Poloxamer 407 display shear thinning characteristics. The G′ values of the three inks were 8 × 10^5^ Pa, 4 × 10^5^ Pa and 3 × 10^5^ Pa for GNP, GO and their mix, respectively, Figure 19b [211]. These storage moduli values are larger compared with those reported for equivalent water-based GNP and GO inks, which were prepared by utilizing polyelectrolytes (anionic and cationic) [212]. The yield stress, which is related to the change of the inks to a semi-liquid state, stays between 1 and 4 kPa. Subsequently, the as-prepared pastes were used for the printing of 3D structures. Next, the structures achieved a very high compressive strength (above 2 MPa) after thermally treated at 1200 °C with a low density (0.12 g/cm^3^) and very high electrical conductivity (above 4 × 10^3^ S/m) for the mix GO–GNP composition. 

The previous examples showed that the classic production of graphene-based ceramic pastes involves the use of various polymers, which are later removed to get a composite of both ceramic and graphene-based materials. Different works have been carried out to develop new formulations and methods of paste preparation to reduce the number of additives in them.

**Figure 20:**
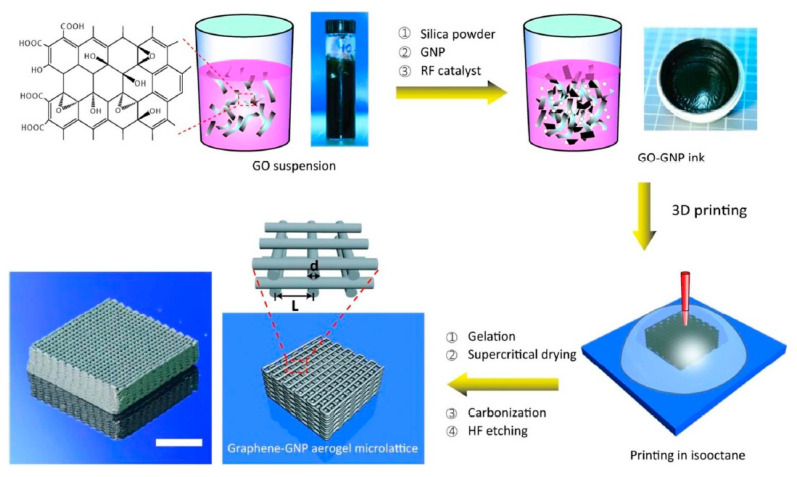
Schematic diagram part fabrication process: Mixing of SiO_2_, GNPs and R-F with the aqueous GO suspension. Then, the as-prepared GO paste was extruded in an isooctane bath, and the as-obtained part was gelled at 85 °C, then dried using supercritical carbon dioxide. Finally, the silica fillers were etched using diluted hydrofluoric acid. The scale bar is 10 mm. Reprinted Reproduced from [213], with permission from American Chemical Society, 2016.

One solution can be to modify the paste rheological behavior to reach suitable viscoelastic characteristics by adding some amount of silica. For example, Zhu et al. investigated the method for manufacturing 3D graphene composite aerogel with periodic macropores for supercapacitor by DIW, Figure 20 [213]. Here, to prepare a suitable paste for DIW the GO suspension (40 mg/mL) was mixed with hydrophilic fumed silica. Silica acted as a viscosifier that imparted both shear-thinning behavior and a shear yield stress to the GO suspension to enhance the printability of the GO-based paste. Besides, the authors added several graphene nanoplatelets (GNPs) along with a reactant (resorcinol–formaldehyde (R–F) solution) to induce gelation post-printing via organic sol-gel chemistry. GNPs and SiO_2_ concentrations ranged from 0 to 16.7 wt% for both materials. The results demonstrated that the apparent viscosity of as-prepared composite paste (GO–GNP) shows orders of magnitude higher than that of the GO suspension; moreover, both of them were shear-thinning non-Newtonian fluids. The presence of the GNP and silica fillers in the pure graphene oxide ink has led to improved storage modulus and yield stress more than one order of magnitude. The magnitudes of these main rheological characteristics coincide with those stated for other colloidal inks fabricated for DIW. In order to obtain the 3D graphene aerogel (GA) the printed composite was subjected to gelation, freeze-drying or supercritical-drying, and etching of the silica with hydrofluoric acid. Although in this work efforts were made to avoid the addition of polymer additives, the inclusion of silica did not completely solve this problem, since a reactant was still used for gelation of the paste.

Another approach to preparing pastes with appropriate rheological properties for ceramic/graphene composites manufacturing by DIW could be the use of preceramic polymers (PCP) [214]. Preceramic polymers are polymeric compositions, particularly as organosilicon compounds (e.g., polymers based on a Si backbone containing N, O, H, C, and B atoms), which under pyrolysis at above ~800 °C in an atmosphere of argon or nitrogen are transformed into ceramic materials, also referred to as polymer derived ceramics (PDCs) [215]. With the addition of PCPs into the graphene-based feedstock is possible to alter the properties, structures and phase of the material after heat treatment.

**Figure 21:**
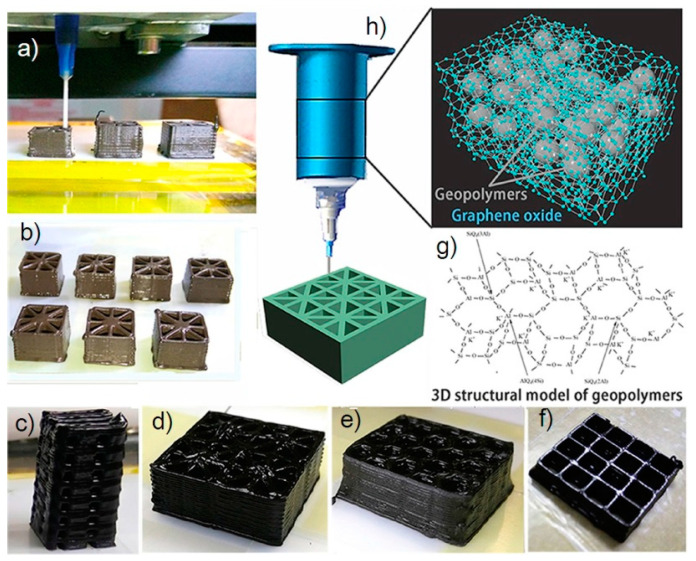
(**a**–**f**) 3D printing process and some 3D printed structures. (**b**–**f**) The colors of the printed samples turn from brownish to blackish when the GO loading increased. (**g**) The chemical structure of geopolymer, and (**h**) schematic diagrams of the painting process and the composite structure are also showed. Reproduced from [209], with permission from Elsevier, 2017.

**Figure 22:**
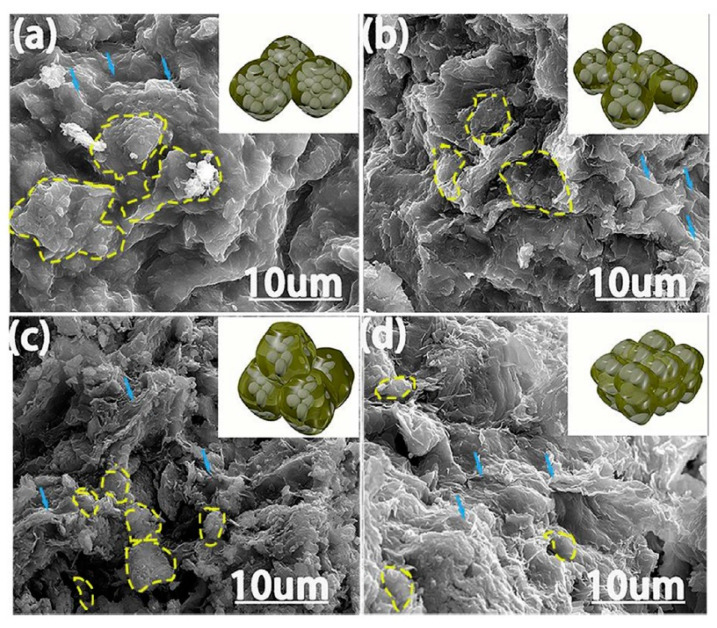
SEM images of hydrated geopolymer particles encapsulated by graphene oxides sheets (**a**–**d**), and their models. With the increase of GO concentration from 4 wt% to 20 wt% in nanocomposites, the agglomerate size (showed by dotted-line circles) decrease. Reproduced from [209], with permission from Elsevier, 2017.

Pierin et al. reported a method for the manufacturing of micro-sized SiOC ceramic components by DIW using a preceramic polymer [216]. The mixing of siloxane resin dissolved in a solvent with cross-linked preceramic grains ensured the appropriate rheological performance of pastes. Moreover, for improved the structural stability via pyrolysis the low amount (0.025–0.1 wt%) of GO was added to the paste formulation, resulting in reduced shrinkage of the preceramic polymer. The resulting parts after pyrolysis at 1000 °C showed an appropriate value of 2.5 MPa and 3.1 MPa of compression strength for a 64 vol% total porosity and after the addition of 0.1 wt% GO, respectively. Zhong et al. first developed GO/geopolymer (GOGP) nanocomposite structures fabricated by DIW, Figure 21 [209]. The authors noted that the addition of graphene oxide in the geo-polymeric water-based mixture (aluminosilicate and alkaline-source particles) intensely modifies its rheology behavior allowing the DIW which would not be possible solely by geo-polymer. Paste preparation involves the obtaining of geo-polymeric suspension by mixing of alkaline-source particles and aluminosilicates particles (ASOPs) in water. After stirring for 20 min, suspensions with diverse amounts of GO (4, 5, 10, and 20 wt%) were added into the as-prepared geo-polymeric suspension at a temperature below 5 °C. This low temperature avoids the geo-polymerization and the GO reduction that could happen at relatively high temperatures which in turn can lead to heterogeneous structure due to agglomeration of nanoparticles. When GO is added into geo-polymeric suspension, its rheological properties change dramatically. For the GOGP with 4 wt% of graphene oxide the storage (G′) and loss modulus (G″) increased to ~1 × 10^5^ Pa and ~1.5 × 10^4^ Pa (at the stress of 50 Pa, that is typically used in DIW) that are over one and two orders of magnitude higher than the values of storage (G′) and loss (G″) moduli of pure geopolymer, respectively. In addition, the yield stress of the GO-based geo-polymeric suspension is as high as ~2000 Pa. When the GO concentration increases up to 5 wt% of GO the yield stress decrease to ~1000 Pa, while the storage modulus increased further. However, when the concentration of GO in nanocomposites increases above the range of 10, and 20 wt%, a decrease of the modulus is showed, which is probably associated with the lubrication effects of GO. The characterization of cured parts showed that GO nanosheets anchored themselves in geo-polymer and encapsulated individual geo-polymer grains, Figure 22 [209], in order to obtain a 3D network across the nanocomposites. The as-obtained cured parts showed high mechanical properties (compressive strength > 30 MPa), while after sintering at 1000 °C the parts achieved a conductivity of 10^2^ S/m.

In the state of the art of graphene-based ceramic 3D printed composites using preceramic polymers, interesting methods, which differ from the above examples in the way that ceramic phase is introduced in the 3D part, can be found. Commonly in these solutions, a 3D graphene-based part is first manufactured by DIW; then, it is heated for polymer removal followed by an infiltration step of a ceramic precursor [217,218,219].

**Figure 23:**
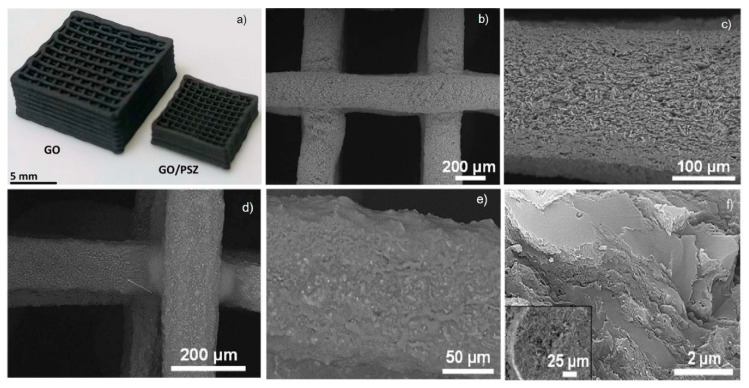
(**a**) 3D printed scaffolds of GO (as-printed) and the composite structure GO/PSZ pyrolyzed at 800 °C. SEM micrographs of a GO lattice after dying/lyophilization steps showing a top view (**b**) and the surface of an extruded filament (**c**). Analogous SEM images of a PSZ infiltrated GO lattice pyrolyzed at 800 °C in N_2_, (**d**) top view, (**e**) filament and (**f**) cross-section at different magnifications. Reproduced from [217], with permission from Elsevier, 2018.

Román-Manso et al. developed an approach to manufacture PDC/GO composites. In this low-temperature method, the first 3D structures were fabricated by DIW using an aqueous GO paste with polymeric additives. Then, the obtained graphene oxide periodic structures were dried in a drying furnace at ~80 °C and immediately afterward frozen in a refrigerator at −20 °C. This leads to prevent the formation of a network of evenly spaced cracks in the composites structure caused by the presence of water. Subsequently, the as-fabricated graphene oxide structures were lyophilized to sublimate the ice. Finally, in order to ensure the diffusion of the liquid into the structure rods, highly porous 3D structures were impregnated by immersion in a liquid organic-polysilazane (a compound of Si, C, H, N) during several hours. For crosslinking and pyrolysis these impregnated structures were placed on the Pt foil in alumina crucibles in a tubular electric furnace and heated at 200 °C and 800–1000 °C, respectively, in N_2_ atmosphere. Figure 23a [217], shows the printed graphene oxide and pyrolyzed composite structure which has remained the shape retention and the high shrinkage.

Figure 23b,c, [217], exhibits views from above of a sublimated GO structure at different magnifications. The linear shrinkage of the lattice was caused by quick-drying treatment.

Figure 23d–f, [217], show printed GO structures after the complete infiltration. No substantial cracking (Figure 23d,e, [217]) is detected in the infiltrated structures after pyrolysis (800–1000 °C). These PDC/GO composites imitate modeled graphene oxide skeleton and, while the conductive network (electrical conductivity in the range 0.2–4 S/cm) of the composite is provided by the presence of graphene. The ceramic coating serves as a protective barrier for the graphene network against the atmosphere, temperature (up to 900 °C in the air) and even direct flame. 

Similar work was carried out by Moyano et al. [218], in which they studied the electrical, mechanical and capacitive responses of a strong and light 3D ceramic/graphene structure obtained through a controllable and fast infiltration method using a preceramic polymer.

Another interesting approach was reported by You et al. [219]. In this work, the authors proposed a method for the growth of SiC that it exactly occurs in 3D printed graphene scaffolds by means of chemical vapor infiltration (CVI). The structures were fabricated using the addition of graphene to ethylene glycol butylether (EGB) in ethanol, followed by sonication and addition of dibutyl phthalate (DBP) and polyvinyl butyral (PVB), resulting in homogeneous graphene-based suspension. Next, the ethanol was evaporated in a water bath at 80 °C with continuous stirring. The as-prepared suspension had a graphene concentration of 200 mg/mL. After, the graphene scaffolds were printed using the as-prepared paste. Subsequently, the printed objects were located in the Ar flow through a carbon tube furnace and heated to 1100 °C for thermal decomposition of organic-polymer. The polymer decomposition allows a large specific surface area of the scaffold that has a positive effect on the densification and the in-situ growth of the SiC. Thus, the SiC matrix was introduced into the pores of the 3D graphene scaffold by cracking methyltrichlorosilane (MTS) in the CVI process. The concentration and structure of the SiC in the composite were monitored by adjusting the holding time and gas pressure, which are the main CVI parameters. 

Finally, the 3D graphene/SiC composites show enhanced mechanical properties, especially compressive strength (193 ± 15.7 MPa) which is 394% higher compared to directly mixed products. Besides, the reconciling of the 3D graphene structure and SiC matrix produces a huge number of conductive paths and gives a composite improved electrical conductivity compared to traditional ceramic materials.

Unfortunately, in these last three examples, it is not possible to directly obtain a 3D printed part from which a graphene-based/ceramic composite is obtained after sintering. In these examples, an additional step of ceramic material infiltration into the graphene skeleton is necessary.

As we have seen in this section, each of the discussed methods includes a preparing step of the graphene-based paste that requires at least the presence of an additive, which is mainly utilized to guarantee a homogeneous dispersion and to achieve the suitable viscoelastic properties. In the majority of cases, the additives are eliminated either by a chemical etching or a thermal process at high temperatures, which causes the appearance of pores in its structure that can negatively influence the composite mechanical properties.

In recent years, attempts have been made to find new methods to minimize the presence of additives in graphene-based paste formulations for DIW. For example, García-Tuñón et al. developed a clean, flexible and robust approach to formulating pastes used in DIW that can be adapted to a wide range of materials [29]. Thus, they prepared free additive pastes of diverse materials (polymer, ceramic and metal), based only on the use of GO as the dispersant, rheological modifier, and binder. This procedure was possible to realize thanks to the great similarities between GO and clay. These materials have a flake-like shape with oxygen-containing functional groups on their basal planes and the edges that promote the network connection between particles thanks to the electrostatic and noncovalent interactions for clay and GO respectively. Clay has exceptional chemistry and structure that permit the design of water-based suspensions for shaping with excellent viscoelastic behavior. For this reason, it is added to ceramics suspensions to reach the required viscoelastic behavior for processing. On the other hand, as for clay, the especial combination of GO sheets surface chemistry and structure in contact with water under special conditions allow the preparation of a very stable GO paste with proper viscoelastic behavior for different materials with have a broad variety of particle morphologies, sizes, and chemistries. In this research, various kinds of graphene-based paste with ceramic (Al_2_O_3_ powders and platelets, SiC powders) were prepared and, then, used for the printing of ceramic parts. The increases of the paste concentration were reached by two different approaches: 1–redispersing freeze-dried GO powders and 2–by evaporation of water at 70 °C. In the two cases, pastes with a high concentration of GO and the necessary viscoelastic behavior for printing were obtained without the addition of any additive. Furthermore, in some cases of the paste preparations, certain amounts of freeze-dried GO powder were added in order to achieve the necessary characteristics of viscoelasticity and flow. Finally, the pastes prepared in this work were formed as indicated below: (1) 28.4 vol% SiC with 0.4 vol% GO (10 mg/mL), (2) 23 vol% Al_2_O_3_ platelets (0.8 vol% GO (23 mg/mL)) and (3) 27 vol% Al_2_O_3_ platelets (1.1 vol% GO (33 mg/mL)).

**Figure 24:**
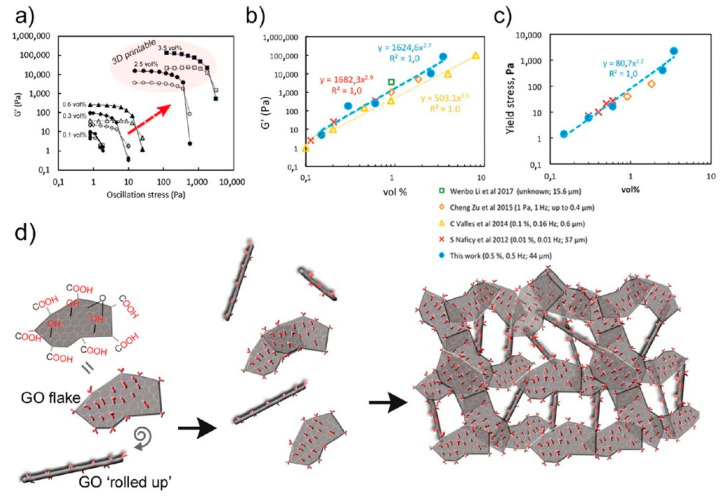
Viscoelastic behavior of pastes and GO suspensions: Storage Modulus (G′) vs. Oscillation Stress (**a**), and GO concentration influence on G′ (**b**), and yield stress (**c**). The proposed network created by GO flakes as concentration increases (**d**). A part of GO sheets form GO scrolls that together with the sheets bring together forming a 3D liquid crystal structure with high G′ (**a**). Reproduced from [29], with permission from American Chemical Society, 2017.

The authors found that GO suspensions with a concentration above ~2 vol% showed an increase of the storage modulus (G′, Figure 24a,b, [29]) as a result of a well-established and organized formed structure in them (Figure 24d, [29]). The reorganization of the GO flakes occurs when the concentration of the suspension increases thus creating a network like a liquid crystal, which produces growth of the storage modulus (G′) up to 100 kPa (Figure 24b, [29]) and the yield stress up to 2300 Pa (Figure 24c, [29]). Besides, the GO pastes with concentrations from 2.5 to 3.5 vol% have the necessary structure and rheological properties for DIW (labeled 3D-printable in Figure 24a, [29]).

**Figure 25:**
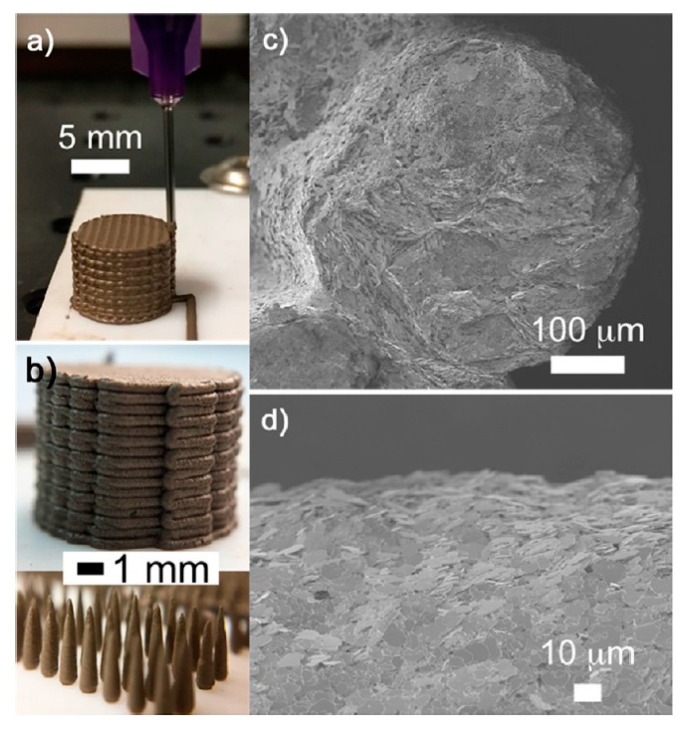
Printed objects from GO/Al_2_O_3_ platelets paste (**a**,**b**) and cross-section and lateral view of printed filament (**c**,**d**). Reproduced from [29], with permission from American Chemical Society, 2017.

**Figure 26:**
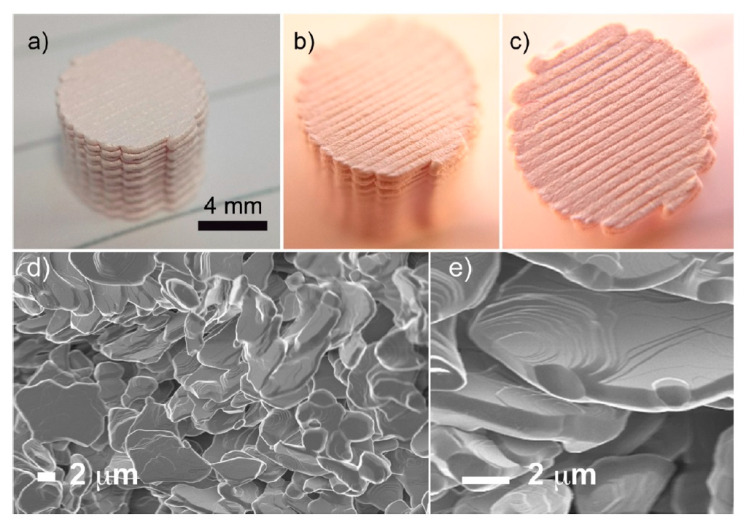
Cylinder printed by DIW from GO/Al_2_O_3_ platelets paste and sintered at 1550 °C. (**a**–**c**). The SEM images show the cylinder microstructure with open porosity of 60% determined by Archimedes’ Principle (**d**,**e**). Reproduced from [29], with permission from American Chemical Society, 2017.

For the Al_2_O_3_ platelets pastes, the concentration of 1.1 vol% GO and ~28 vol% platelets showed the greatest behavior for printing (Figure 25, [29]). During extrusion an orientation of platelets and an internal structure formation of printed filaments took place. The FESEM images of printed filament cross-section and lateral view are shown in Figure 25c,d, [29]) respectively. In them is possible to see how the platelets form a wall on the outside edge (Figure 25d, [29]), while the filament inside part has a mixture of domains (Figure 25c, [29]). A more detailed observation demonstrated that GO sheets are distributed over and across multiple Al_2_O_3_ platelets interacting with a very strong form, binding them together and forming bridges across them. After sintering, the structures made with GO had an average porosity of 60% with only 2% closed pores and showed good handling strength, Figure 26 [29].

The SiC paste also showed suitable printing behavior. Dried 3D printed bars had strengths of ~1 MPa which demonstrates that in this case the GO also operates as a binding agent between the SiC particles. Bars printed from GO/SiC paste and sintered at 2050 °C for 2 h showed a density of 3.21 g/cm^3^ and reached a bending strength of around 212 MPa.

In both cases, Al_2_O_3_ and SiC were the unique crystalline phase in the sintered objects, and Raman spectroscopy demonstrated that no carbon residues remained in the structure. Note that, it is possible to add potentially structural or functional properties to the sintered 3D object simply retaining the GO in the structure after sintering

Summarizing, this method permits us to form complex 3D ceramic structures using DIW, which have properties that are similar to alternative formulations, and demonstrates the possibility of using 2D colloids in materials manufacturing.

## 5. Summary

In this review, it was shown that significant advances in additive technologies for 3D printing of graphene-based ceramic composites have been made in recent years. The state of the art of different additive techniques used for the manufacturing of both ceramic and graphene-based pieces was analyzed. In addition, various examples of 3D printing of graphene-based ceramic composites were discussed in detail.

First, a summary of existing additive technologies groups, techniques that are involved with them, and of the most popular feedstock nowadays was made, (see Table 1). After that, it was clear that not all AM technologies apply to the ceramic part manufacturing and even more for the graphene-based materials.

The introduction of Additive Manufacturing to the production of ceramics is related to the need to obtain complex parts that are not possible to produce using conventional methods since 3D printing can manufacture complex structures in a fast, simple and inexpensive way. With the aim of a better understanding, the AM technologies used in ceramic production were divided into three groups (powder-, slurry- and bulk solid-based) taking into account the type of feedstock used. The state of the art of AM technologies that are involved with these groups has been considered in detail and demonstrated with symbolic examples. Moreover, several historical facts about each technique were given.

The techniques (SLS, SLM, and BJ) are involved in the group of powder-based technologies for the manufacture of 3D ceramic parts. SLS and SLM have a low surface finish, undesirable porosity, and high shrinkage of parts that limit their application in many fields. Besides, the thermal gradients and the high heating and cooling rates in the ceramic material produce cracks and distortions that are not desired in the final part. On the other hand, in BJ the formation of pores and the contraction of parts are related to the elimination of the binder used in the process. In BJ the mechanical properties of parts are affected by pore formation, despite this, this technique is a good method used in biomedical fields for the ceramic scaffold production.

The liquid-based group includes the SLA, DLP, TPP and IJP techniques. These methods proved to be more promising than the powder-based ones as they can achieve high resolution, good surface finish and required mechanical properties. Unfortunately, the high cost of the machines and the photopolymers that are necessary for the manufacture of parts limit their wide application in the industry. Furthermore, the working principle of TPP allows only the use of transparent polymers; i.e., the opaque polymers that were usually utilized in SLA and DLP are not applicable for TPP. On the other hand, IJP is limited only to the production of miniaturized parts due to the low ink volume used in each droplet.

In the third group are LOM, FDM and DIW techniques. In recent years, great development of the LOM technique has not been observed and its application has been restricted only for the manufacture of ceramic parts with simple geometry, and large sizes. On the contrary, FDM and DIW have found a great application in the manufacture of macro-pore structures for the biomedicine application as the production of scaffolds.

AM techniques have also been used for 3D printing of graphene-based materials thanks to the combination of its properties with the advantages of slurry-based methods and techniques that use a pseudoplastic feedstock. Additionally, the choice of such technologies is related to the fact that graphene oxide, the main precursor of graphene, can be easily dispersed in water and other solvents, which is not possible with graphene. Among the most used methods for printing graphene-based material, we can find SLA, IJP, FDM, and DIW techniques.

The SLA method has been utilized to manufacture polymer-based composites that are used in the production of scaffolds in biomedicine. On the other hand, IJP is one of the most used techniques in the printing of graphene-based materials despite the low resolution and limitations that it presents. It is possible to found polymer-based filaments with graphene fillers for FDM that are used in the production of parts for biomedicine or in electronics and other areas of application. The disadvantage of this method is its low precision and the quality of the surface.

DIW is the most studied technique and one of the most widely used for the manufacture of 3D parts from a graphene-based feedstock thanks to the combination of the great possibilities of DIW with the unique properties of graphene that has demonstrated remarkable printing capabilities with unique viscoelastic properties. For DIW, the rheology of the suspension is very important, so it is essential to establish the appropriate content of the components to obtain their homogeneous dispersion. After the part is printed, a subsequent process is necessary for the removal of the solvent, which leads to the appearance of pores in the structure. Similar structures are widely used in the production of biomaterial scaffolds and energy storage fields.

Finally, a detailed study with some examples of printed composites from graphene-based ceramic pastes by DIW is carried out. In this field, there is a tendency to reduce the number of additives that are used for obtaining a homogeneous dispersion and very often produce undesired effects. Some examples show that it is possible the use preceramic polymers for the reduction of additives and to perform the characteristics of printed composites. Of great importance is the work carried out to create ceramic pastes without any other additives than graphene oxide. Here it is appreciated how this material can be used as a dispersant, rheological modifier and binder at the same time.

This work has tried to show that the DIW method is very promising in the printing of complex graphene-based ceramic composites at reduced cost and in less time. We consider that this field should continue to develop so that soon the manufacture of graphene-based ceramic composites will take its place in the ceramic industry.

## Figures and Tables

**Table 1:** Groups of additive manufacturing technologies by the International Organization for Standardization (ISO)/American Society for Testing Materials (ASTM) 52900:2015.

Category	Additive Manufacturing Technology Type	Abbreviation	Feedstock
Vat photopolymerization	Stereolithography	SLA	Liquid photopolymers, hybrid polymer-ceramic, hybrid polymer-graphene
	Digital Light Processing	DLP	
	Two-Photon Polymerization	TPP	
	Continuous Liquid Interface Production	CLIP	Liquid photopolymers
Powder bed fusion	Multi Jet Fusion	MJF	Thermoplastic polymers
	Selective Laser Sintering	SLS	Plastics, composites
	Selective Laser Melting	SLM	Metals
	Electron Beam Melting	EBM	Metals
Material jetting	Material Jetting	MJ	Photopolymers
	NanoParticle Jetting	NPJ	Metals, ceramics
	Drop On Demand	DOD	Wax, ceramic-, graphene-inks
Material Extrusion	Fused Deposition Modeling	FDM	Thermoplastic polymers, metal-, ceramic-, graphene-reinforced polymers
	Direct Ink Writing	DIW	Ceramics
Direct Energy Deposition	Electron Beam Additive Manufacturing	EBAM	Metals and alloys in the form of powder or wire
	Laser Engineering Net Shape	LENS	
Binder jetting	Binder Jetting	BJ	Ceramic, metal, gypsum, sand
Sheet Lamination	Laminated Object Manufacturing	LOM	Ceramic, metal-filled tapes, paper, polymer composites.
